# Evidence of shared transcriptomic dysregulation of HNRNPU-related disorder between human organoids and embryonic mice

**DOI:** 10.1016/j.isci.2022.105797

**Published:** 2022-12-10

**Authors:** Andrew K. Ressler, Gabriela L.A. Sampaio, Sarah A. Dugger, Tamar Sapir, Daniel Krizay, Michael J. Boland, Orly Reiner, David B. Goldstein

**Affiliations:** 1 Institute for Genomic Medicine, Columbia University Irving Medical Center, New York, NY 10032, USA; 2 Department of Genetics and Development, Columbia University Irving Medical Center, New York, NY 10032, USA; 3 Department of Neurology, Columbia University, New York, NY 10032, USA; 4 Department of Molecular Genetics, Weizmann Institute of Science, Rehovot, Israel; 5 Incumbent of the Berstein-Mason Professorial Chair of Neurochemistry, Head of M. Judith Ruth Institute of Preclinical Brain Research, Weizmann Institute of Science, Rehovot, Israel

**Keywords:** Psychiatry, Biological sciences, Developmental biology, Embryology, Omics, Transcriptomics

## Abstract

Generating effective therapies for neurodevelopmental disorders has remained elusive. An emerging drug discovery approach for neurodevelopmental disorders is to characterize transcriptome-wide dysregulation in an appropriate model system and screen therapeutics based on their capacity to restore functionally relevant expression patterns. We characterized transcriptomic dysregulation in a human model of *HNRNPU*-related disorder to explore the potential of such a paradigm. We identified widespread dysregulation in functionally relevant pathways and then compared dysregulation in a human model to transcriptomic differences in embryonic and perinatal mice to determine whether dysregulation in an *in vitro* human model is partially replicated in an *in vivo* model of *HNRNPU*-related disorder. Strikingly, we find enrichment of co-dysregulation between 45-day-old human organoids and embryonic, but not perinatal, mice from distinct models of *HNRNPU*-related disorder. Thus, hnRNPU deficient human organoids may only be suitable to model transcriptional dysregulation in certain cell types within a specific developmental time window.

## Highlights

• *HNRNPU*^*+/−*^ hCOs are significantly smaller than wild-type organoids
• *HNRNPU*^*+/−*^ hCOs show increased number of dysregulated genes in precursor populations
• Transcriptomic dysregulation in *HNRNPU*^*+/−*^ hCOs concordant with embryonic mice
• Embryonic and perinatal *Hnrnpu* mutant cortices do not show consistent dysregulation

## Graphical abstract

**Figure:**
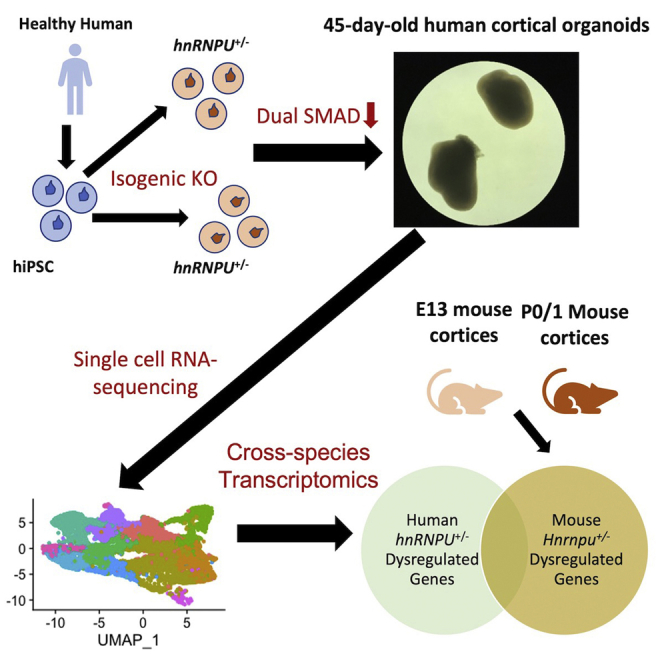

## Introduction

Despite the enthusiasm often expressed about precision medicine approaches in neurodevelopmental disorders,^1^ the development of mechanistically targeted therapies remains highly challenging. Indeed, despite remarkable progress in identifying genetic causes underlying such disorders,^2^^,^^3^^,^^4^ only a handful of genetic neurodevelopmental disorders are currently treated with effective, targeted therapies. There are likely to be many reasons for this slow progress, but two in particular stand out. First, implicated genes are highly heterogeneous, implying the necessity for distinct drug discovery paradigms for different genetic disorders. Second, it is often unclear what model system is most suitable for finding and testing targeted treatments.

We systematically explore both of these challenges by focusing on the neurodevelopmental disorder caused by variants in *HNRNPU*. *De novo* loss-of-function variants in *HNRNPU* have been found to cause a neurodevelopmental syndrome, with patients exhibiting developmental delay (∼95%), intellectual disability (52%), craniofacial dysmorphism (54–95%) including microcephaly (14–20%) and seizures (83–95%).^5^^,^^6^^,^^7^hnRNPU is expressed throughout development and has been implicated in abnormal cardiac development^8^ and regulates expression of the prominent developmental morphogen Shh.^9^ Furthermore, hnRNPU is an RNA- and DNA-binding protein^10^^,^^11^^,^^12^ found to be involved in diverse processes including alternative splicing,^8^^,^^13^ chromatin remodeling^14^ and telomere shortening.^15^

Recently, Dhindsa et al. argued that neurodevelopmental disorder genes, such as *HNRPNU*, that appear to cause disease through widespread dysregulation of the transcriptome or epitranscriptome might be treatable through an approach that uses small molecules to move genome wide expression patterns back toward the normal state.^16^ Because hundreds of genes that function as transcription factors, RNA binding proteins or chromatin remodelers^17^^,^^18^^,^^19^^,^^20^ cause neurodevelopmental disorders, optimization of such a paradigm might have wide applicability. However, key questions about this approach remain unresolved, including identifying appropriate model systems capable of characterizing the nature of transcriptomic dysregulation and to test the relationship between transcriptomic restoration and functional improvement.

Here, we address these challenges through the characterization of transcriptomic dysregulation in an organoid model of *HNRNPU*-related disorder and by comparing differential expression in organoid models to mouse models of *HNRNPU*-related disorder. These comparisons allow us to assess for the first time the developmental nature of transcriptomic dysregulation in *HNRNPU*-related disorder and the extent to which patterns of dysregulation in brain organoids are reproducible in an *in vivo* model system. While supporting some aspects of the paradigm outlined by Dhindsa et al., this work shows that the challenges to be overcome are more substantial than were initially apparent.

## Results

### Generation of *HNRNPU*^+/−^ stem cell lines

**Figure 1:**
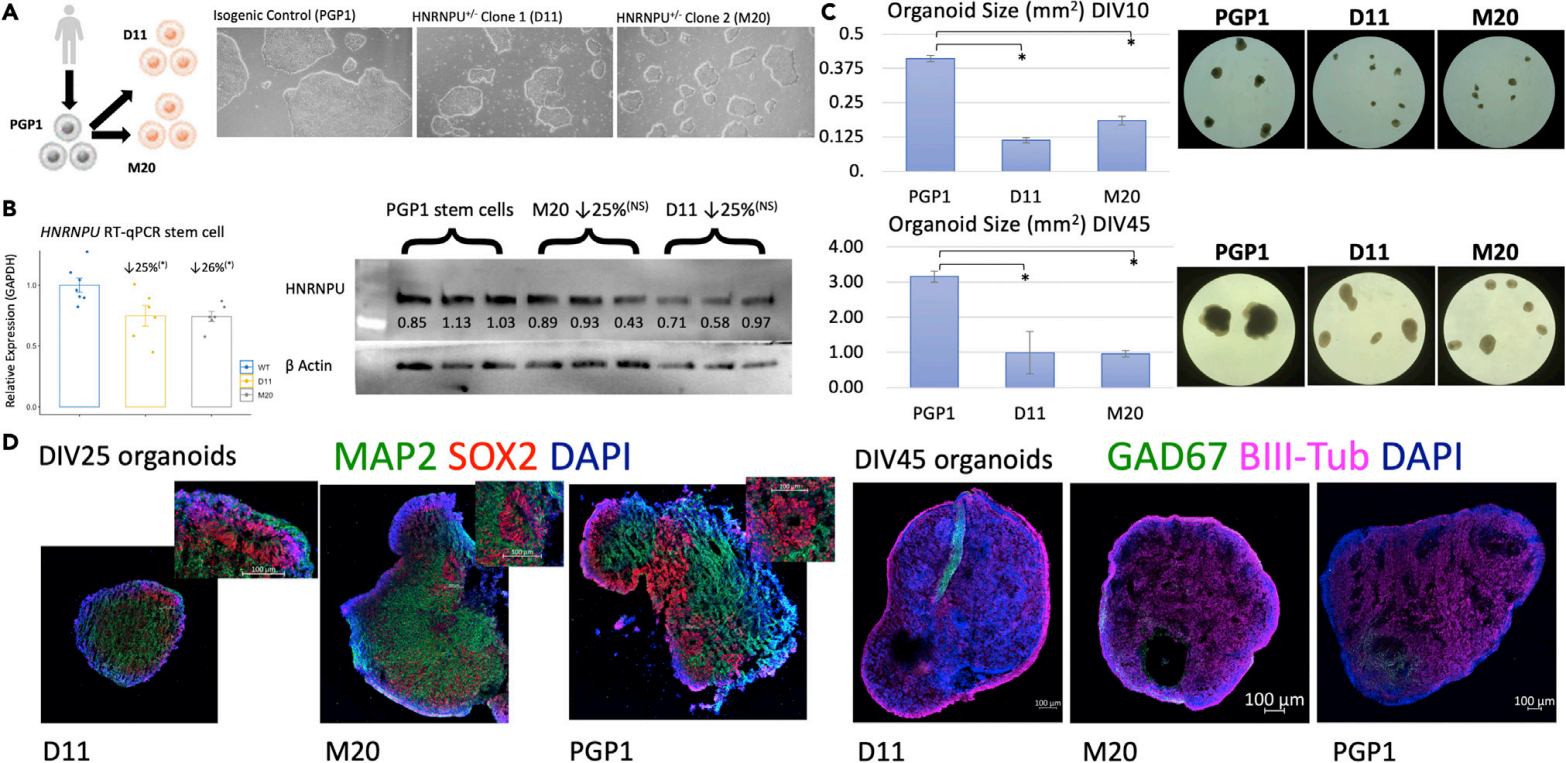
*HNRNPU*^+/−^ stem cell lines successfully differentiate into human cortical organoids with significant size reduction (A) Two isogenic *HNRNPU*^+/−^mutant stem cell lines were generated by SynthegoTM and representative phase contrast images of stem cell populations are shown. (B) Both *HNRNPU*^+/−^ stem cell lines show approximately 25% reduction in hnRNPU mRNA (left) and protein (right) expression compared to internal controls. ∗ = p< 0.05. NS = not statistically significant. (C) *HNRNPU*^+/−^ hCOs show significant size reduction emerging by DIV10 and continuing through 45 days *in vitro*. ∗ = p< 0.05. (D) D11, M20 and PGP1 exhibit VZ-like proliferative zones at DIV25 (left) and widespread neuronal expression (right) at DIV45 with evidence of subpopulations of GABAergic neurons. Data are represented as mean ±SEM.

To characterize transcriptomic dysregulation in a human model of *HNRNPU*-related disorder, we first generated two isogenic mutant lines using the publicly available PGP1 induced pluripotent stem cell (iPSC) line (RRID:CVCL_F182) with a 1 bp duplication (‘D11’) and a 10 bp deletion (‘M20’), respectively (Figure 1). Mutations in D11 and M20 result in a frameshift and a premature stop-codon in exons 2 and 4 respectively (Figure S1). Importantly, both D11 and M20 stem cells exhibited a significant reduction in hnRNPU mRNA expression, alongside an ∼25% reduction in protein levels (Figure 1).

### *HNRNPU*^+/−^ cortical organoids show consistent transcriptomic dysregulation and size difference versus isogenic controls

We next generated human *HNRNPU*^+/−^ cortical organoids (‘hCOs’) as described previously^21^ and similarly identified VZ-like regions in 25-day-old hCOs generated from PGP1, D11 and M20 hiPSCs (Figure 1). By day 45, hCOs widely expressed BIII-tubulin, a neuronal marker, and additionally included GABAergic subpopulations (Figure 1). Although all stem cell lines were capable of generating hCOs, both *HNRNPU*^+/−^mutant lines generated significantly smaller organoids, with a size phenotype emerging as early as DIV 10 and maintaining throughout the ∼6.5-week long experiment (Figure 1). The reduction in size of hCOs may phenocopy the subset of human patients with microcephaly. Furthermore, size phenotypes have been identified in distinct mouse models of *HNRNPU*-related disorder. An *Hnrnpu* heterozygous knockout mouse model with a constitutive 113 base pair deletion in exon 1 results in a significant size reduction in mice, with proportional cortical thickness.^22^ Similarly, a distinct constitutive heterozygous mutation that generates a truncated protein shows a similar overall size reduction in mutant mice.^23^ Finally, the same truncating mutation, specifically disrupted in Cre expressing cells (*Emx1*^*Cre*^), results in significant reduction in cortical size in homozygous mice.^24^

Once we confirmed *HNRNPU*^+/−^ hCOs successfully differentiated into appropriate neuronal populations and displayed a size difference consistent with a subset of patient and mouse model phenotypes, we aimed to identify a robust signature of transcriptomic dysregulation in mutant hCOs. After 45 days *in vitro*, cortical organoids were dissociated into single cells and sequenced. To help ensure the transcriptomic signature was robust, we sequenced a total of 42 organoids per line, with five biological replicates from three experiments in two batches of single-cell RNA (‘scRNA’) sequencing. Using pseudobulk analyses from scRNA-seq samples, we found significant overlap of both upregulated and downregulated genes across batches and across mutant cell lines, with higher replication of DEGs between batches (Figure S2).

### *HNRNPU*^+/−^ organoids show disruption in neurodevelopmental processes and ribosomal proteins

**Figure 2:**
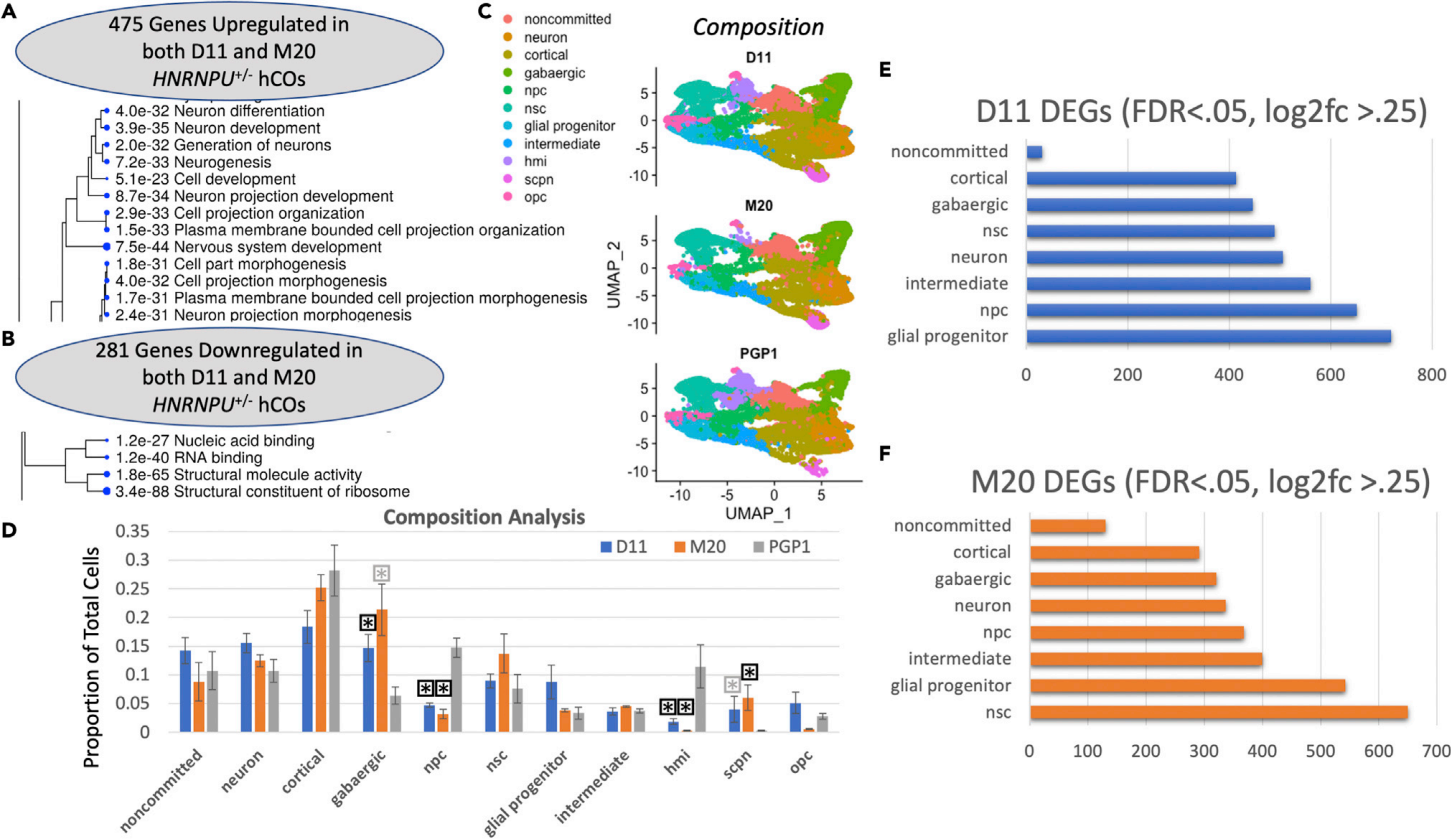
*HNRNPU*^+/−^ hCOs show disruption in ribosomal proteins and neurodevelopmental processes (A) Upregulated genes in *HNRNPU*^+/−^ hCOs enriched for neurodevelopmental ontologies, including neurogenesis, morphogenesis and differentiation. (B) Downregulated genes enriched for functional annotations including DNA-binding and RNA-binding. (C) UMAP visualization of annotated clusters across all five samples for D11, M20 and PGP1. ‘npc’ = neuronal progenitor. ‘nsc’ = neural stem cell. ‘hmi’ = high metabolic intermediate progenitor. ‘scpn’ = sub-cortical projection neuron. ’opc’ = oligodendrocyte precursor. (D) Compositional analysis shows significant reduction (∗ = p< 0.05) in NPC and HMI populations for both D11 and M20 hCOs. Significant or trending significant (∗ = p< 0.06) increases in SCPN and GABAergic populations for both D11 and M20 hCOs. (E and F) show burden of dysregulated genes increased in precursor populations versus more mature neuronal population. Number of DEGs generated using 47 cells per cluster per sample to control for cell number and sample bias. FDR-correct pvalues and a log_2_ fold change threshold of 0.25 were used. Final DEGs averaged across 10 simulations to minimize impact of selection bias. Precursor populations were the four most (M20) or four of the five most (D11) impacted populations. Data are represented as mean ±SEM.

We then further characterized the patterns of dysregulation seen in *HNRNPU*^*+/−*^ hCOs at both pseudobulk and cell-type specific resolution. When characterizing transcriptomic dysregulation, we chose to prioritize specificity over sensitivity of dysregulated genes and determined genes to be differentially expressed if they were significantly enriched in both D11 and M20 organoids (log_2_fc >0.25, FDR<0.05). Using this relatively stringent approach, we identified 281 downregulated and 475 upregulated genes. Importantly, downregulated genes were most heavily enriched for ribosomal proteins and genes associated with molecular functions including nucleic acid binding and RNA binding, both reported as prominent functions of hnRNPU.^12^^,^^25^^,^^26^ Similarly, upregulated genes were heavily enriched for neurogenic and neurodevelopmental processes (Figure 2A) with several genes implicated in neurodevelopmental disorders present in the top 18 upregulated DEGs (log_2_fc>1) including *NR2F1*,^27^ *SYT1*^28^ and *GAD1*.^29^ Thus, patterns of dysregulation in an organoid model of *HNRNPU*-related disorder are directionally consistent with an RNA-binding protein driven neurodevelopmental disorder.

Of interest, three members of the hnRNP gene family, *SYNCRIP*, *HNRNPD*, and *HNRNPM* are included amongst the 475 upregulated genes (Table S1). A recent meta-analysis from Gillentine et al.^6^ elucidates overlap in function and structure across hnRNP family genes and specifically identified a strong positive correlation in expression patterns between hnRNPD and hnRNPU in the developing brain, implicating potential genetic redundancies that may modulate disease etiology. In the same meta-analysis, Gillentine et al. identified hnRNP family genes were most consistently upregulated in dividing radial glia. *HNRNPU* was further found to have increased expression in radial glia and progenitors of the medial ganglionic eminence, two additional immature cell populations. We thus hypothesized our organoid model may show increased burden of dysregulation in dividing and less mature cell populations.

### *HNRNPU*^+/−^ hCOs show compositional differences and differential burden of DEGs between precursor populations and neurons

Even within several weeks *in vitro*, hCOs include a heterogeneous population of cortical cell types.^21^ Furthermore, Dugger et al., recently identified increased burden of dysregulation in a specific subpopulation of cells in a mouse model of Hnrnpu haploinsufficiency.^22^ Thus, we aimed to further characterize transcriptomic dysregulation in an organoid model at single-cell resolution. We first used the Seurat pipeline^30^ to synchronize all samples into a single shared space and annotated clusters using a combination of canonical markers and GO ontologies.^31^^,^^32^

Briefly, we first identified neuronal populations by expression of *DCX*, *STMN2* and *GAP43*. Within the neuronal populations, one cluster did not show enrichment of GABAergic markers or excitatory neuronal markers and was labeled as non-committed neuron. Remaining clusters were considered GABAergic (*GAD1*, *GAD2*), cortical projection neurons (*NEUROD2*, *NEUROD6*), SCPNs (*FOXP2*, *NTNG2*, and *RORB*) or neuron (GO: Glutamate secretion).

Neural clusters without significant populations of neurons (*VIM*, *NES*, *HES1*) were annotated as NSCs, NPCs, intermediate progenitors, high-metabolism intermediate progenitors (‘HMIs’), glial progenitors and OPCs using GO ontologies for upregulated genes in specific clusters (Figure S3). D11, M20 and PGP1 organoids generated cells from all 11 populations (Figure 2B). We then confirmed expression of *HNRNPU* in all cellular subtypes and found the highest average expression was in neural stem cells (Figure S4), consistent with results in the developing fetal brain that showed increased expression in dividing cell populations.^6^

We then investigated whether or not both mutant cell lines, D11 and M20, showed consistent disruption in generating certain cell populations (Figure 2C, Table S2). Here, the 5 organoid samples for each line were considered as independent replicates and we assessed compositional differences for each mutant line to ensure aberrant population proportions were robust. We found consistent reduction in NPCs and HMIs in both mutant clones. Similarly, GABAergic and SCPN populations were enriched in one mutant line with corresponding borderline significance (p< 0.06) in the second mutant clone. Importantly, reduced populations were evident in progenitor and intermediate populations, whereas enriched populations were in committed neuronal populations.

Finally, we considered whether or not certain cell populations were more heavily burdened in D11 and M20 organoids. We restricted our analyses to clusters with greater than 10 cells in all 15 samples, which led to the exclusion of HMI, SCPN and OPC populations. All remaining clusters had at least 47 cells in every cluster. Thus, we downsampled each cluster in every sample to 47 cells to ensure burden analyses were not driven by sample bias. For both D11 and M20, precursor populations (NSCs, glial progenitors, NPCs and intermediate progenitors) tended to be more heavily burdened than neuronal populations (non-committed, cortical, GABAergic and neuron). Of interest, both compositional analyses and burden analysis are in line with findings in *Hnrnpu*^*fl*/fl^ mice showing instability and death in neuronal progenitor populations (*Emx1*^*Cre*^) where *Hnrnpu* is conditionally deleted.^24^

Thus, *HNRNPU*^+/−^ hCOs show a characteristic pattern of dysregulation shared across isogenic mutant lines, including evidence of cell-type specific burden of dysregulated genes, with more immature cell types exhibiting increased burden of disease. For either global or cell-type specific dysregulation of *HNRNPU*^+/−^ hCOs to be suitable for potential therapeutic reversal, such dysregulation must be scalable to an *in vivo* system. Because characterizing transcriptomic dysregulation in the brains of patients is intractable, we instead consider whether or not transcriptomic dysregulation in a human model is partially reproduced *in vivo* using mouse models of *HNRNPU*-related disorder.

### Transcriptomic dysregulation of *HNRNPU*^+/−^ cortical organoids replicated in E13 cortices

Although attempts at broad transcriptomic or epitranscriptomic comparisons of a human and mouse model of neurodevelopmental disease are exceedingly rare, the few existing studies suggest consideration of developmental time point may be necessary to identify concordant dysregulation across model systems (Figure S5). Recent pseudotime analyses of hCOs generated similarly to those described herein suggest that around 6.5-week-old hCOs correspond closest to fetal human brains around post-conception week (‘pcw’) 10.^33^ Furthermore, a recent single-cell transcriptomic analysis of mouse neocortex found that E14.5 mouse cortices most closely resemble humans between 7 and 11.5 pcw, whereas P0 mice are most analogous to pcw 20–23 in fetal samples.^34^ Thus, we first compared the dysregulation seen in *HNRNPU*^+/−^ hCOs to embryonic (E13) mouse cortices.

We initially considered two distinct mouse models for investigation, a constitutive knockout and a conditional truncating mutation in a subset of neocortical progenitors (*Emx1*^Cre/+^). Bulk-RNA sequencing for E13 heterozygous “*Hnrnpu*^*fl*/-^” and homozygous “*Hnrnpu*^*fl*/fl^” cortices has previously been analyzed,^24^ whereas constitutive “*Hnrnpu*^+/−^” embryonic cortices were dissected and sequenced. Importantly, whereas initial characterization of hCOs express populations of GABAergic neurons at 72 and 79 days *in vitro*, such organoids were found to recapitulate dorsal cortical development,^21^ suggesting hCOs may be enriched for excitatory neuronal processes. Thus, we did not have initial assumptions as to which model may better recapitulate transcriptomic dysregulation seen in cortical organoids.

When using a simple threshold of FDR-adjusted pvalues <0.05 following differential expression analysis with DESeq2, we identified a large number of DEGs (n = 2,802) for *Hnrnpu*^fl/-^ embryonic mice and relatively few for time matched *Hnrnpu*^+/−^ cortices (n = 172). Furthermore, *Hnrnpu*^fl/-^ samples clearly co-clustered in PC space, whereas *Hnrnpu*^+/−^ samples were not distinguishable from WT littermates and segregated instead by gender (Figure S6). We thus prioritized the *Hnrnpu*^*fl*/-^ for cross-species comparisons given the stronger evidence of widespread dysregulation and a larger number of DEGs to assess.

**Figure 3:**
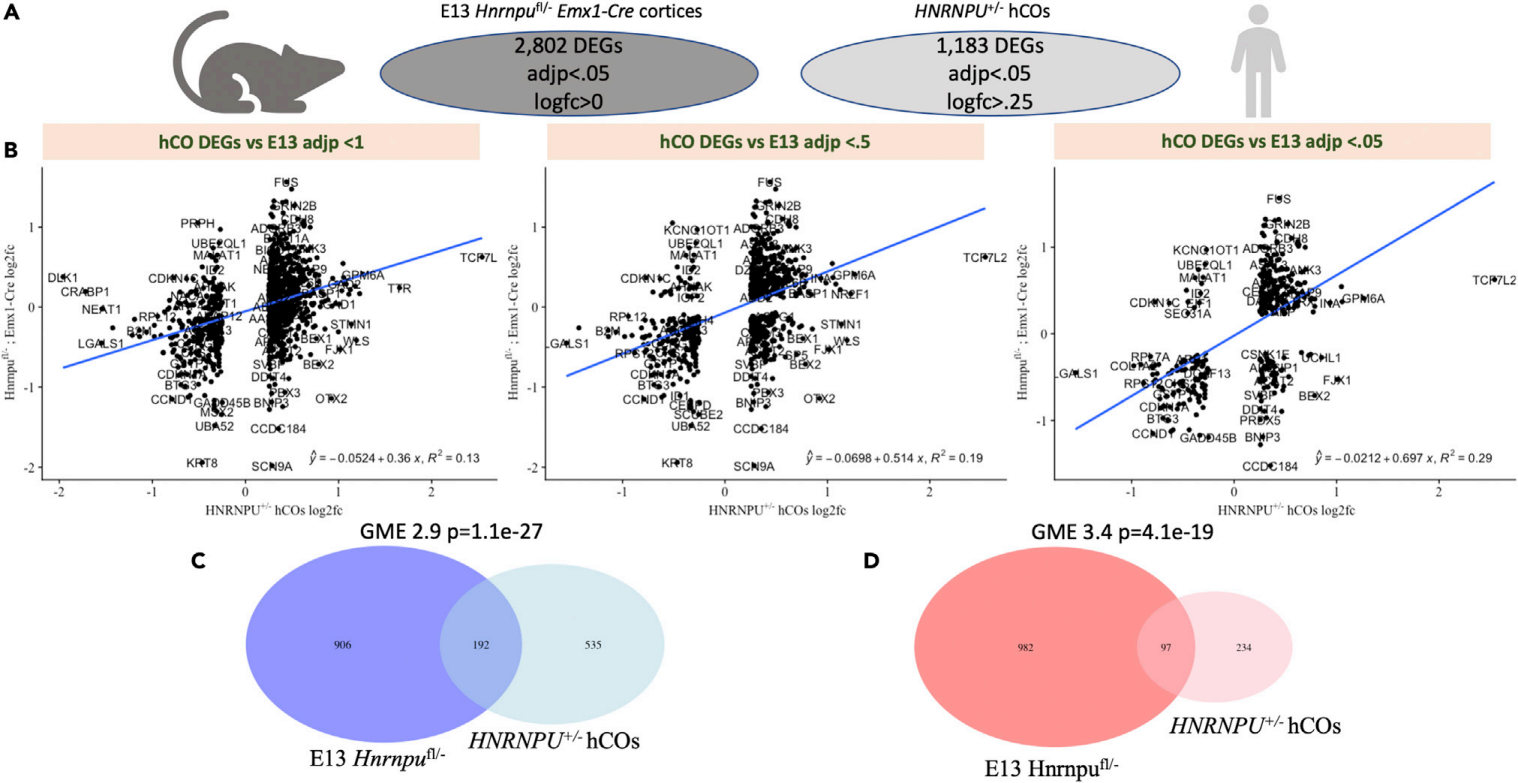
Consistent dysregulation between DIV45 *HNRNPU*^+/−^ hCOs and E13 *Hnrnpu*^*fl/-*^ mouse cortices (A) Overview of differentially expressed genes (DEGs) in E13 cortices and hCOs. (B) Log_2_ fold change of all hCO DEGs were plotted against log_2_ fold change values of correlate genes in E13 *Hnrnpu*^*fl/-*^ mouse cortices, without consideration of whether or not those changes were identified as differentially expressed in E13 samples (left). A best fit linear regression line was plotted and resulted in an R^2^ coefficient of 0.13. Same analysis was performed considering only genes that were both differentially expressed in hCOs and FDR adjusted significance thresholds in mice of either p<0.5 (middle) or p<0.05 (right). When considering genes with FDR<0.05 in both models, R^2^ coefficient increases to 0.29. Quanitification of geometric mean enrichment further shows significant enrichment of both co-upregulated (C) and co-downregulated (D) genes.

For cross-species comparisons, we considered all differentially expressed genes (‘DEGs’) identified in hCOs across 5 samples per line, with D11 and M20 treated as biological replicates for *HNRNPU*-related disorder. Thus, a single log fold change value for each gene differentially expressed in *HNRNPU*^+/−^ hCOs can be generated. To assess whether or not transcriptomic dysregulation in a human *in vitro* system is reproduced *in vivo*, we first plotted the log fold change of all hCO pseudobulk DEGs versus the log fold change of their mouse correlates in bulk E13 RNA sequencing of *Hnrnpu*^*fl*/-^ cortices and wild-type littermates. Of interest, even without any log fold change or significance thresholds for E13 mice, there is evidence of recapitulation of hCO signature, with a modest R^2^ of 0.13. With increasingly stringent FDR-adjusted pvalue thresholds, the correlation increases up to an R^2^ of 0.29 (Figure 3B). Importantly, such correlation is seen across species, mutations and subtype specificity, with selective reduction of *Hnrnpu* in *Emx1*^*Cre*^ cells, suggesting such cross-species comparisons can be robust.

Once we identified positive correlation of the transcriptomic signature seen in hCOs, we quantified the overlap of FDR-adjusted DEGs (log_2_fc > 0.25) between *HNRNPU*^+/−^ hCOs (n = 1,183, 796 upregulated, 387 downregulated) and *Hnrnpu*^*fl*/-^ cortices (n = 2,802, 1,519 upregulated, 1,283 downregulated). We found highly significant enrichment in genes both similarly upregulated and similarly downregulated, without any enrichment of genes dysregulated in opposing directions (Figures 3C and 3D; Table S3). Reassuringly, we identify more modest, but consistent patterns between hCOs and *Hnrnpu*^+/−^ embryonic cortices, with an R^2^ coefficient of 0.31 for log_2_fc of DEGs and 6.2-fold enrichment of downregulated genes without any corresponding enrichment of discordant gene sets (Figure S7A).

### Transcriptomic dysregulation of perinatal mice discordant with *HNRNPU*^+/−^ cortical organoids and E13 mice

**Figure 4:**
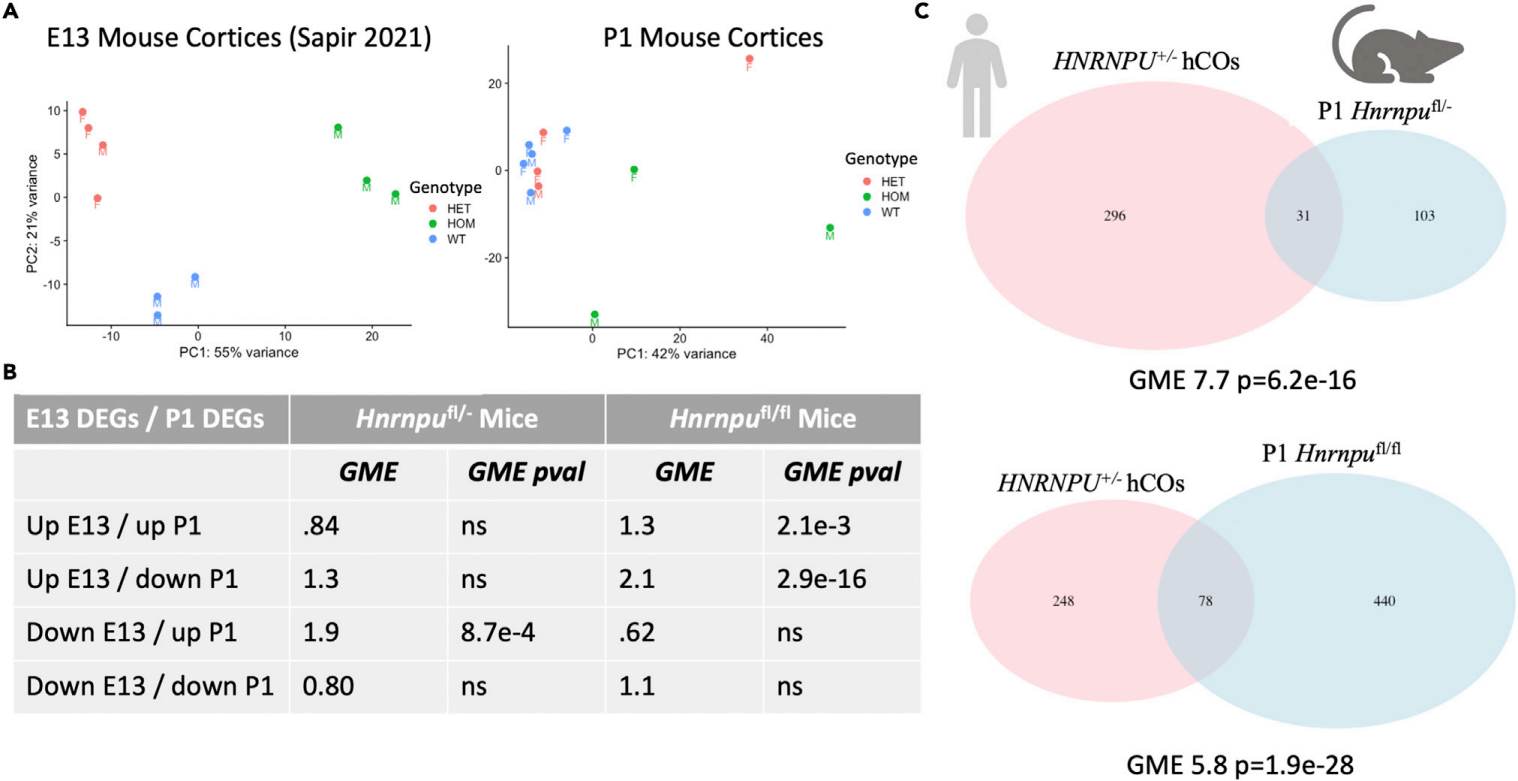
Transcriptomic signature of *HNRNPU*-related disorder diverges in perinatal mice (A) Evidence of reduced transcriptomic dysregulation in perinatal *Hnrnpu*^*fl/-*^ cortices. Both heterozygous and homozygous mice segregate in PC space in RNA-sequencing of embryonic cortices, but heterozygous samples do not segregate at a perinatal time point. “F” = female. “M” = male. (B) Both *Hnrnpu*^*fl/-*^ and *Hnrnpu*^*fl/fl*^ cortices show most significant geometric mean enrichment is in genes differentially expressed in opposing directions at embryonic and perinatal time points. (C) Downregulated genes in DIV45 *HNRNPU*^+/−^ hCOs are significantly enriched in gene sets that were identified as upregulated in both *Hnrnpu*^*fl/-*^ and *Hnrnpu*^*fl/fl*^ P1 cortices.

Paradigms of transcriptomic reversal rely on patterns of dysregulation to be recapitulated in pediatric patients and transcriptomic dysregulation may not necessarily be static throughout development. Given the extended timelines of human neurodevelopment compared to mice^35^ and the partial reproduction of *HNRNPU*^+/−^ disease signature in embryonic mice, we examine a perinatal murine time point to address whether or not transcriptomic signatures of *HNRNPU*-related disorder may be stable. We first asked whether or not the transcriptomic dysregulation seen in embryonic mice was reproduced perinatally. To this end, we considered the dysregulation of both *Hnrnpu*^*fl*/-^ and *Hnrnpu*^*fl*/fl^ mice at an embryonic and perinatal time point. Of interest, although both heterozygous and homozygous genotypes cleanly clustered together at embryonic time points, perinatal heterozygous samples predominantly co-clustered with matched wildtype samples (Figure 4A), suggesting more limited disruption later in development.

We then examined whether or not significantly dysregulated genes at perinatal time points were consistent with embryonic mice. Because of the limited overall disruption in heterozygous mice, unadjusted pvalues were used to determine significant genes in those samples, whereas FDR-adjusted pvalues were considered for homozygotes. Surprisingly, the most significant finding for both mouse models was enrichment of discordant gene sets (Figure 4B).

We similarly assessed whether dysregulation of *HNRNPU*^+/−^ hCOs was similarly discordant with perinatal mice. We found genes downregulated in *HNRNPU*^+/−^ hCOs were significantly upregulated in *Hnrnpu*^*fl*/-^ mice. Similarly, whereas *HNRNPU*^+/−^ hCOs and *Hnrnpu*^*fl*/fl^ mice showed enrichment of co-upregulated and co-downregulated genes at an embryonic time point, the strongest enrichment at a perinatal time point was once again upregulation of genes downregulated in hCOs. Furthermore, when considering the log_2_fc of DEGs in organoids versus DEGs in perinatal *Hnrnpu*^*fl*/-^ and *Hnrnpu*^*fl*/fl^ samples, we found evidence of modest transcript level anti-correlation (R^2^ of 0.19 and 0.10 respectively, Figure S7).

Finally, we examined perinatal *Hnrnpu*^+/−^P0 cortices to see whether the same directional effects were present and similarly found modest anti-correlation (R^2^ of 0.15, Figure S8B). Although there was no evidence of concordant or discordant gene sets, we examined whether or not the genes discordant between hCOs and perinatal *Hnrnpu*^*fl*/-^ cortices may be recapitulated, but underpowered. Significantly, 27 of the 31 discordant genes showed raw increases in log_2_fc, suggesting such genes are similarly upregulated in newborn *Hnrnpu*^+/−^ mice (Figure S8C). Of interest, discordant genes are enriched for ribosomal proteins, in addition to including genes such as Cdkn1a and Eif3m, which have been found to be involved in cell-cycle arrest^36^ and organ size,^37^ respectively.

In summary, *HNRNPU*^+/−^ hCOs reproduce significant aspects of transcriptomic disruption in embryonic mice but are strikingly discordant with perinatal samples. Importantly, compositional differences may underlie a portion of the discordance of dysregulated genes between perinatal cortices and hCOs and embryonic samples. Several glial populations emerge from Emx1-expressing precursors^38^ and gliogenesis emerges around E16.5.^39^ Glial populations may have distinct patterns of dysregulation driven by *HNRNPU*-related disorder and may contribute to the discordance of dysregulation across developmental time points. Furthermore, no mouse model of *HNRNPU*-related disorder to date has shown evidence of spontaneous seizures, with *Hnrnpu*^+/−^ mice exhibiting a reduced seizure threshold and homozygotes were nonviable.^22^ Lack of existing evidence of spontaneous seizures, alongside transcriptome-wide co-clustering of *Hnrnpu*^+/−^ and *Hnrnpu*^*fl*/-^ mice with their respective wild-type controls may suggest a more modest phenotype in mice than in genotypically matched patients.

## Discussion

Within this work we aimed to address whether or not human organoid systems can be utilized in therapeutic stratagems to characterize and reverse transcriptomic dysregulation driven by the large number of genes that may cause neurodevelopmental disorders through widespread dysregulation of the transcriptome. We begin to explore this fundamental question by first characterizing dysregulation in a human cortical organoid model of *HNRNPU*-related disorder. We first identified modest reduction (∼25%) in hnRNPU expression consistent with a previously described constitutive mouse model. Furthermore, *HNRNPU*^+/−^ hCOs resulted in a significant size reduction, consistent with both evidence of microcephaly in a subset of patients and size reduction across several mouse models of *HNRNPU*-related disorder. After identifying recapitulation of basic features of *HNRNPU*-related disorder in a human model, we examined transcriptional dysregulation at global and cell-type specific resolutions.

Importantly, human brain organoid systems are known to suffer from high levels of variability, amongst other concerns about how faithfully brain organoid systems recapitulate aspects of the fetal brain.^40^ To account for inter-organoid and experimental batch variability, we utilized a total of 42 organoids across three different experiments and two different sequencing for two isogenic *HNRNPU*^+/−^ stem cell lines for transcriptomic analyses. Promisingly, we were able to identify strong consistency of DEGs in 6.5-week-old *HNRNPU*^+/−^ hCOs across independent differentiations and distinct isogenic mutant lines (Figure S2). When considering only genes differentially expressed in both isogenic lines, we identified widespread dysregulation of the transcriptome with downregulated genes enriched for gene ontologies consistent with the known functions of hnRNPU (nucleic acid binding, RNA binding). Furthermore, upregulated genes were enriched for neurodevelopmental processes and several genes implicated in neurodevelopmental disorders were amongst the most upregulated.

A significant outstanding question in transcriptomic reversal therapeutic paradigms is whether to consider cell-type specific or more global transcriptomic dysregulation. Thus, we further characterized *HNRNPU*-related disorder in hCOs at single-cell resolution. First, we performed compositional analyses and identified significant reductions in progenitor populations, (hmi and npc) alongside corresponding increases in certain more mature neuronal populations (GABAergic and scpn). We then identified a trend of increased burden in precursor populations versus more mature neurons, suggesting transcriptomic signatures may be sensitive to cell-type. Importantly, we only considered a singular developmental time point. Although 6.5-week-old organoids are heterogeneous, the neuronal populations remain highly immature. A recent report suggests human brain organoids are capable of generating cell types more similar to postnatal populations, but maturation timelines of at least 250–300 days are required.^41^ Characterizing transcriptomic dysregulation in significantly older organoids would be necessary to properly characterize the presence or absence of cell-type specific dysregulation in a human system.

Alhtough *HNRNPU*^+/−^ hCOs show evidence of widespread transcriptomic dysregulation, whether or not an *in vitro* system is scalable to *in vivo* disease is critically unclear. Species-specific transcriptomic differences are likely to exist between humans and mice for all transcriptional regulators involved in neurodevelopmental disorders, including hnRNPU, given highly distinct developmental timelines and brain complexity. However, in certain instances, significant elements of dysregulation may be conserved and mouse models and be used to validate human *in vitro* systems are scalable to an *in vivo* system. Within this work, we demonstrate transcriptome level dysregulation in a human organoid system is not predominantly driven by *in vitro* artifacts, as disease signature shows enrichment of co-dysregulated genes with embryonic mouse models.

Critically, the transcriptomic signature of *HNRNPU*-related disorder in 6.5-week-old human organoids is not reproduced in mice at perinatal time points. Such divergent dysregulation is consistent with assessing the developmental trajectory of differential expression exclusively in mouse systems. In fact, in both Hnrnpu^fl/-^ and Hnrnpu^fl/fl^ mice, the most significantly enriched gene sets are genes with opposing direction. Thus, transcriptomic signatures of *HNRNPU*-related disorder are highly confounded by developmental time point and considering the maturity of model system is absolutely essential when developing therapeutics for patients. Furthermore, therapeutic paradigms focused on mouse models alone may suffer from more modest phenotypes in heterozygous mice compared to human patients. Future studies focused on understanding functional differences of hnRNPU in humans and mice may better contextualize transcriptomic findings and improve our understanding of pathogenesis across species.

Importantly, as the brain develops, celltype composition is variable, with certain populations of cells, such as glia, emerging later in neurodevelopment. Performing single-cell sequencing at several developmental time points would allow for a more robust assessment of whether transcriptomic differences are specific to cellular ages and/or subtypes.

All models considered within this manuscript are driven by loss-of-function variants, consistent with the majority of known pathogenic variants for *HNRNPU*-related disorder.^6^ However, the mutations introduced are not patient-specific and transcriptomic dysregulation may vary significantly for certain variants. Particularly, the subset of pathogenic missense variants in *HNRNPU* without a known direction of effect may not be effectively modeled by the loss-of-function models within this manuscript.

In total, we have shown an immature organoid model of *HNRNPU*-related disorder has dysregulation consistent with embryonic time points, but not perinatal time points. Furthermore, even though we only considered a single time point, we were able to identify preliminary evidence of developmental differences in transcriptional signature in a human model, with precursor populations exhibiting increased burden of dysregulated genes compared to more mature neuronal populations. However, to robustly investigate both developmental and cell-type specificity of dysregulation in a human model of *HNRNPU*-related disorder, a time course of single-cell RNA sequencing with organoids up to hundreds of days old may be required. Mouse models of *HNRNPU*-related disorder may also be confounded by cell-type specificity and single-cell sequencing across time points may elucidate whether certain cell populations, including both neuronal and glial subtypes, have patterns of dysregulation that are more stable over certain developmental time windows. Such analyses in mouse and human systems could be used to determine if and when the transcriptomic signature of *HNRNPU*-related disorder may begin to stabilize.

### Limitations of the study

Although transcriptomic approaches to disease modeling are valuable; we believe functional phenotypes will be essential to developing effective therapeutics for *HNRNPU-*related disorder.

Therefore, we believe the most significant limitation of the work within this manuscript, in addition to previously mentioned limitations related to organoid variability, developmental time points assessed and nature of specifc genetic perturbations in human and mouse models, is the absence of any electrophysiological analyses comparing hCOs and mouse models. Unfortunately, hCOs undergo protracted developmental timelines, in line with human neurodevelopment21; thus, we did not attempt to identify electrophysiological phenotypes in *HNRNPU*^+/−^ hCOs given the extended timelines necessary to generate functionally mature neuronal networks in line with even the embryonic mice. We believe this is a limitation shared across organoid models, as generating mature networks capable of identifying seizures in a human *in vitro* model of any epileptic encephalopathy has remained elusive.^42^ Without robust functional phenotypes in human *in vitro* models, a cross-species approach may be suitable. For example, a compound of interest may be screened based on ability to reverse transcriptomic dysregulation in a human model. Such a compound may theoretically be tested for ability to reduce functional phenotypes in a mouse model. A compound capable of reversing transcriptomic dysregulation in a human model alongside activity phenotypes in an *in vivo* mouse model may be more robust and result in improved patient outcomes. Such an approach may be critically limited by more muted electrophysiological phenotypes in certain mouse models, but potentially would still improve on existing paradigms.

*HNRNPU* is just one of hundreds of genes that may cause a neurodevelopmental disorder through widespread perturbation of the transcriptome and alternative genetic disorders may have more stable transcriptomic signatures. Nevertheless, this work underscores concerning limitations of therapeutic paradigms relying on transcriptional reversal. Specifically, inappropriate therapeutic reversal of gene expression based on dysregulation in immature human organoids may actually exacerbate patient phenotypes in instances where disease signature is partially reversed at later developmental stages.

## STAR★Methods

### Key resources table

**Table:** 

REAGENT or RESOURCE	SOURCE	IDENTIFIER
**Antibodies**		

Rabbit monoclonal against N-terminus	Abcam	ab180952
HRP-conjugated b-Actin	Santa Cruz	sc-47778
MAP2 (mouse)	R&D Systems	MAB8304-SP
SOX2 (rabbit)	Cell Signaling Technology	mAb #3579
GAD67 (mouse)	Synaptic Systems	198,211
BIII tubulin (rabbit)	Abcam	ab107216

**Experimental models: Organisms/strains**		

Emx1-Cre B6.129S2-Emx1tm1(Cre) Krj/J	The Jackson Laboratory	No: 005628,
UBC-Cre-ERT2 B6.Cg-*Ndor1*^*Tg(UBC−Cre/ERT2)1Ejb*^/1J	The Jackson Laboratory	No: 007001
Hnrnpu^+/113DEL^	Columbia University Irving Medical Center (Institute for Genomic Medicine)	Dugger et al., bioRxiv

**Experimental models: Cell lines**		

PGP1	Synthego	PGP:hu43860C
D11	Synthego	Available upon request
M20	Synthego	Available upon request

**Chemicals, peptides, and recombinant proteins**		

DMEM F-12	Caisson Labs	DFL15
Knockout serum replacement	Thermo Fisher	10828–028
MEM-NEAA	Thermo Fisher	1114005
Glutamax supplement	Thermo Fisher	35050061
β-Mercaptoethanol	Sigma	M7522
LDN-193189	Sigma	SML0559
SB431542	Reprocel	400100
XAV939	Stemgent	040,046) 2
Neurobasal medium	Thermo Fisher	12348017
Insulin	Santa Cruz	sc-360248
Penicillin/Streptomycin	Caisson Labs	PSL01
N2 supplement	ThermoFisher	17,502,048
B27 supplement without vitamin A	ThermoFisher	12,587,010
B27 supplement	ThermoFisher	17,504,044
BDNF	R&D Systems	248-BDB-050
Ascorbic acid	Tocris	4055
Tissue-Tek O.C.T	Sakura	4538
paraformaldehyde	ThermoFisher	28,906
Triton X-100	ThermoFisher	85,111
Geltrex	ThermoFisher	A1413201
mTeSR Plus	StemCell	100-0276_C
EDTA	Invitrogen	AM9260G
U-bottom ultra-low-attachment 96-well plate	Corning	CLS7007-24 EA
ProLongTM Diamond Antifade Mountant with DAPI	ThermoFisher	P36961
NuPAGE Bis-Tris	ThermoFisher	NP0326BOX
Xcell II Blot Module to 0.2	ThermoFisher	EI9051
Amersham ECL reagents	Cytiva	RPN2232
EBSS	ThermoFisher	24,010,043

**Software and algorithms**		

bcl12fastq2 (version 2.19)	Illumina	1,000,000,035,330
kallisto (0.44.0)	https://pachterlab.github.io/kallisto/	https://doi.org/10.1038/nbt.3519
DESeq2	https://bioconductor.org/packages/release/bioc/html/DESeq2.html	https://doi.org/10.1186/s13059-014-0550-8
Seurat v3	https://satijalab.org/seurat/	https://doi.org/10.1016/j.cell.2021.04.048
ShinyGO^43^ software	http://bioinformatics.sdstate.edu/go/	https://doi.org/10.5281/zenodo.1451847

**Equipment**		

NanoDrop 2000	ThermoFisher	ND-2000C
Zeiss Axio Observer Z1 Fluorescence Motorized Microscope	Zeiss	4,565,235
Xcell II Blot Module	Thermo Fisher	EI9051
QuantStudioTM 5	Applied Biosystems	A34322
Bioanalyzer	Agilent	G2939A
Illumina NovaSeq 6000	Illumina	20,023,471
Echo Revolve microscope	Echo	Revolve

**Datadeposited**		

Human Single Cell RNA-sequencing data (raw and processed) and Murine RNA-sequencing data (raw and process)	GEO	GSE219317
Hnrnpu^fl/-^& Hnrnpu^fl/fl^ E13 RNA-sequencing data	GEO	GSE181527

### Resource availability

#### Lead contact

Further information and requests for resources and reagents should be directed to and will be fulfilled by the lead contact, Andrew K. Ressler (akr2151@cumc.columbia.edu).

#### Materials availability

For conditional *Hnrnpu* lines, the conditional *Hnrnpu* allele received from Prof. Maniatis,^8^ while Emx1-Cre was obtained from The Jackson Laboratory (Emx1-Cre B6.129S2-Emx1tm1(Cre)Krj/J Stock No: 005,628). For constitutive knockout mouse lines, mice were generated by The Jackson Laboratory, but are not publicly available. The lines are cryopreserved at Columbia University and requests for use will be fulfilled by the lead contact.

Human cell lines D11 and M20 were generated by Synthego by editing the founder PGP1 cell line (PGP1-SV1). For use of D11 and M20 cell lines, requests will be fulfilled contingent on availability of frozen stocks by the lead contact.

### Experimental model and subject details

#### Mouse husbandry and genotyping

*Hnrnpu*^+/−^ mice were generated by The Jackson Laboratory via a CRISPR-induced 113-bp deletion in exon I (Dugger 2020), while*Hnrnpu*^fl/-^ and *Hnrnpu*^fl/fl^ were generated by specific deletion of a genomic DNA region encoding *Hnrnpu* exons 4–14 (Ye 2015) in Emx1 expressing cells. For genotyping, DNA was extracted from tail or ear clippings and genotypes were determined through PCR amplification that generated larger and smaller products for wild-type and mutant alleles, respectively. For *Hnrnpu*^fl/-^ and *Hnrnpu*^fl/fl^ mice, Cre expression was independently confirmed using PCR amplification. All *Hnrnpu*^+/−^ mice had access to regular chow and water and were maintained within the Columbia University Institute of Comparative Medicine, which is fully accredited by the Association for Assessment and Accreditation of Laboratory Animal Care. Similarly, *Hnrnpu*^fl/-^ and *Hnrnpu*^fl/fl^ mice had access to regular chow and water and were maintained within the Weizmann Institute and all animal protocols were approved by the Weizmann Institute Institutional Animal Care And Use Committee.

#### Human induced pluripotent stem cell line generation

Isogenic mutant lines were generated by SynthegoTM from the publicly available PGP1 human induced pluripotent stem cell (‘iPSC’) founder line. This line was obtained from fibroblast tissue of a 55-year-old, caucasian man, generated through retroviral reprogramming. CRISPR-Cas9 gene editing was performed using guide RNA sequence CCACGGCCATGATCTTCTCG, generating a cut site at Chr: 1; 244,862,672, which is within Exon 2 of HNRNPU. Edited clones were expanded and Sanger sequenced, leading to the identification of two isogenic mutant lines with +1 bp (‘D11’) and −10bp (‘M20’) indels respectively. Both D11 and M20 were assessed by Synthego for pluripotency and normal karyotypes using PluriTest and KaryoStat, respectively. Two vials of 1 million cells were then expanded and maintained within Columbia’s Institute for Genomic Medicine (‘IGM’). The iPSCs were maintained in incubators at 37°C and 5% CO_2_, cultured under sterile conditions and split every 3 to 4 days with 5 mM EDTA, and plated on sterile plastic plates coated with GeltrexTM (ThermoFisher) and mTeSR Plus media (StemCell).

### Method details

#### Human cortical organoid differentiation

Human Cortical Organoids (‘hCOs’) were generated using an established protocol.^21^ Briefly, the iPSCs were dissociated into single cells and 9,000 cells were plated per well of a U-bottom ultra-low-attachment 96-well plate (Corning, Cat No. CLS7007-24 EA) in neural induction medium supplemented with 50 μm Rock-inhibitor and 2% FBS. On Day 2, media was replaced with 150 μL neural induction medium supplemented with 50 μm Rock-inhibitor. Media changes every two days with neural induction media was performed until DIV10, when up to 12 embryoid bodies were transferred into each well of an ultra-low-attachment 6-well plate and placed on a shaker set to 225–250 rpm in 2–3 mLs of neural differentiation medium minus Vitamin A. Every two days, media was aspirated and 2–3 mLs of fresh neural differentiation medium minus Vitamin A was added. On day 18, after aspirating wells, neural differentiation medium was added. From day 18 through completion of experiments, media was aspirated, and fresh neural differentiation medium was added every 4–5 days. During the whole experiment, plates were maintained in incubators at 37°C and 5% CO_2_. Media formulations are further described in additional resources.

#### Immunocytochemistry

On days *in vitro* 25 and 45, hCOs were washed 2x with 1X PBS in 500 μL Eppendorf™ tubes. After washing, organoids were fixed for 20–30 min in 4% paraformaldehyde at RT. Following fixation, organoids were washed 3x with PBS and placed in 4°C cold room overnight in 30% sucrose in PBS. Once all fixed organoids descended to the bottom of Eppendorf tubes, they were removed and embedded in Tissue-Tek O.C.T in disposable cryomolds and placed in −80°C freezer until cryosectioning.

Cryosectioning was done using 20 μm slices and up to three slices were placed onto slides for immunocytochemistry. Slides were either returned to the −80°C freezer for future staining or air dried for 30 min at RT. Following air-drying, fixed slices were washed 2x with PBS and then incubated in blocking solution (5% donkey serum and 0.3% Triton X-100 in PBS) for 1–2 h at RT. After blocking, slides were placed in humidified chambers overnight at 4°C in blocking solution supplemented with primary antibodies. The next morning, slides were washed 3X with PBS and then incubated in blocking solution supplemented with Alexa Fluorophore conjugated secondary antibodies for 1–2 h in the dark at RT. Finally, slides were rinsed 4X with PBS and covered by coverslips with ProLong™ Diamond Antifade Mountant with DAPI. After at least 24 h of drying in the dark, Imaging was performed using the Zeiss Axio Observer.Z1 Fluorescence Motorized Microscope and associated Zen2 Pro imaging software. Downstream image processing was performed using PowerPoint, using consistent adjustments to brightness and/or contrast across like images.

#### Western blotting

Stem cells were placed on ice and rinsed 3X with ice-cold PBS. Cells were homogenized using a cell scraper in cold RIPA buffer containing protease and phosphatase inhibitors (Roche). Lysis was completed for 15 min on ice and samples were centrifuged at >8000 RPM for 30 min at 4°C. The resulting supernatant was collected, and protein quantified using BCA method with BSA-based standards. Protein lysates were diluted in LDS sample buffer and Laemmli buffer. Samples were boiled at 95°C and 5 μg was loaded onto a 4–12% Bis-Tris gel in SDS running buffer supplemented with NuPAGE antioxidant and run at 150V for 45 min. Protein was then transferred using the Xcell II Blot Module to 0.2 um methanol-activated PVDF membrane at 30 V for 1.5hat 4°C in Transfer buffer containing 20% methanol. Membranes were then blocked for 1 h in 5% milk at RT and incubated overnight in hnRNPU primary antibody at 1:1000 dilution (Rabbit monoclonal against N-terminus: Abcam ab180952). Blots were developed using Amersham ECL reagents (RPN2105) and imaged with an Azure 300 system. For a loading control, blots were subsequently incubated in an HRP-conjugated b-Actin secondary at 1:1000 (Santa Cruz #sc-47778) diluted in 5% BSA for 1hat RT. Protein expression was quantified using ImageStudioLite with normalization to b-Actin.

#### RT-qPCR

RNA was extracted from stem cells using RNeasy Plus Mini Kit (QIAGEN) and cDNA was generated using Super-Script IV (ThermoFisher) with random hexamers. cDNA was then diluted to approximately 2.5 μg/μL based on quantification of initial RNA concentration using a NanoDrop (ThermoFisher). In each well of a 384-well plate, 10 μg of cDNA (4 μL) was added alongside 5 μL of TaqMan Fast Universal PCR Master Mix 2X and 0.5 μL each of a FAM-conjugated *HNRNPU* TaqMan probe and a VIC-conjugated *GAPDH* probe for an internal control. PCR amplification and quantification of fluorescence was done using a QuantStudio™ 5 Real-Time PCR System. Comparative CT analysis was used and mean and SE of mean bars generated using biological replicates. Each biological replicate generated a single mean value based on 2–3 technical replicates.

#### Bulk RNA-Sequencing

All bulk RNA-sequencing data, generated previously^24^ or newly provided in this manuscript, was generated by manual dissection of cortices from either embryonic (E13) or perinatal mice (P0 or P1), followed by flash freezing the dissociated cortices or incubating in RNAlater prior to RNA extraction at a later time point after all samples in a given experiment had been genotyped. Once mice were genotyped, RNA was generated from relevant cortices using the RNeasy Plus Mini Kit (QIAGEN). To ensure quality of RNA, purity was confirmed using a Bioanalyzer (Instrument Name DE72901373, Firmware C.01.069, Type G2939A). Samples with a RIN score >9 were sent for RNA sequencing. Poly-A pull-down was used to enrich mRNAs from total RNA samples and libraries were constructed using Illumina TruSeq chemistry. Libraries were then sequenced using Illumina NovaSeq 6000 at Columbia Genome Center. RTA (Illumina) was used for base calling and bcl12fastq2 (version 2.19) was used to convert BCL to fastq format, coupled with adaptor trimming. Pseudoalignment was performed to a kallisto index (Mouse: GRCm38) using kallisto (0.44.0).

Differential expression analysis was performed with DESeq2. Differentially expressed genes were identified using DESeq2’s likelihood ratio test (‘LRT’). When genders and litters of mice were distributed across conditions, they were used as latent variables. Genes were considered differentially expressed using FDR <0.05 and no threshold for fold change. For samples with fewer than ten FDR <0.05 DEGs, unadjusted pvalues <0.05 were used to determine DEGs for downstream cross-species or cross-model comparisons (*Hnrnpu*^+/−^P0, *Hnrnpu*^fl/-^ P1 and *Hnrnpu*^fl/fl^ P1).

#### Single-cell RNA-sequencing

hCOs were dissociated into single cells using Papain in EBSS supplemented with DNAse at DIV45-47. Briefly, 6 (experiment 1) or 12 (experiment 2) hCOs per genotype were aggregated together into a 1 mL Eppendorf™ tube and rinsed 2X with PBS then incubated in Papain solution for 30–45 min at 37°C with agitation every 10–15 min. A 1000 μL pipet was then used to triturate organoids into single cells. Cells were then spun down at 0.4 xg for 5 min. Cells were resuspended in PBS with 5% FBS and viability was calculated using Trypan Blue and manual counting on a hemocytometer. Populations with >90% viable cells were placed on ice and brought to JP Sulzberger Columbia Genome Center for sample preparation and single-cell RNA-sequencing.

#### Single-cell RNA-sequencing data analysis

We used Seurat v3 (Stuart 2019) to perform downstream QC and analyses on feature-barcode matrices. First, we removed all genes that were not present in at least 3 cells and removed all cells that did not express at least 200 genes. We then removed all cells with greater than 7500 genes expressed or greater than 10% mitochondrial reads to remove potential doublets and low-quality cells. The filtered matrices were log-normalized and scaled to 10,000 transcripts per cell. We used variance-stabilization transformation to identify the 2,000 most variable genes. We used Seurat’s data integration matrix to synchronize all 15 samples using reciprocal PCA across 3 experiments and 3 lines using 30 dimensions and latent variables mtDNA percentage and reads per cell. Following integration of data, we identified clusters using Seurat’s FindNeighbors (dimensions = 11) and FindClusters functions (resolution = 0.5). We used UMAP reduction and Seurat’s DotPlot and VlnPlot functions to visualize clusters and cell-type specific expression of genes.

To annotate clusters, we used Seurat’s FindConservedMarkers function, which implements a Wilcoxon test and used Bonferroni correction to identify genes enriched in certain clusters. We only considered genes with positive enrichment and expressed in at least 5% of cells. Annotation of clusters was done through a combination of expression of canonical markers visualized with DotPlot function and GO ontologies of most enriched genes (Figure S6).

To identify differentially expressed genes, we used Seurat’s FindMarkers function using MAST with a log fold change threshold of 0.25 and only considered genes expressed in at least 5% of cells. Mitochondrial percentage and read count were used as latent variables. For Pseudobulk analyses, all cells were considered, while cell-type specific analyses were done by subsetting population prior to MAST analysis. A Similar approach was used to identify all Pseudobulk and cell-type specific differentially expressed genes with minimal adjustments. When considering samples from experiment 1 and experiment 2 together, batch was additionally used as a latent variable. For burden analyses, each cluster for each sample was randomly downsampled to 47 cells (n = 235 total cells per line), the minimum number of cells in any of the clusters considered for a given sample. To ensure phenotype was robust to sampling bias, we downsampled and identified differentially expressed genes 10 times and averaged the number of differentially expressed genes across the 10 iterations. Genes were considered differentially expressed with a log_2_fc >0.25 and Benjamini-Hochberg adjusted pvalues (FDR <0.05).

### Quantification and statistical analysis

#### Transcriptomic dysregulation analyses

Pseudobulk analysis of all 5 samples of D11 and M20 combined was compared to PGP1 in cross-species comparisons. Two basic analyses were performed when comparing dysregulation across species, models or developmental time points. First, the log_2_ fold change of all DEGs for *HNRNPU*^+/−^ hCOs were plotted on the xaxis, while the log_2_ fold change of their species-correlate were plotted on yaxis and a best-fit linear regression line with slope and R^2^ coefficient were calculated and displayed. We then show how correlation coefficient is altered when considering only genes that pass certain thresholds of significance in the dataset displayed on yaxis. For all other graphs comparing log_2_fc between models or time points, plots and linear regression lines are generated similarly with only genes significant dysregulated in both datasets being considered. Secondly, we calculate the geometric mean enrichment (‘GME’) of all combinations of upregulated and downregulated genes to quantify whether or not upregulated or downregulated genes are significantly enriched across species, model and time point. Here, we use a one-sided Fisher test on a 2∗2 matrix to calculate GME and associated pvalue. For example, when considering genes upregulated across datasets, the 2∗2 matrix includes genes upregulated in both datasets and genes upregulated in dataset 1 alone in the first row. The second row of input matrix includes genes upregulated in dataset 2 alone followed by all genes not upregulated in dataset 2. Importantly, the number of total genes considered for enrichment analyses is determined by number of genes considered for differential expression testing in both datasets (genes with total reads >10 for bulk-RNA samples and present in 5% of D11/M20 or PGP1 cells for pseudobulk across both experiments were considered). GME fold enrichment and associated pvalue were generated for all combinations of up and downregulated genes. Importantly, for models with fewer than 10 FDR adjusted DEGs, unadjusted pvalue of 0.05 is used for all the analyses described above.

#### Size quantification of hCOs

Phase contrast images of hCOs were taken at DIVs 10 and 45 on an Echo Revolve microscope with 2X magnification. Using the revolve software on an iPad, the borders of hCOs were drawn with an accompanying stylus and area was quantified.

#### Gene ontologies

Gene ontology analysis was performed using ShinyGO^43^ software (http://bioinformatics.sdstate.edu/go/). Genes upregulated and downregulated in same direction in both D11 and M20 hCOs were queried for enrichment of biological processes and molecular function. All upregulated and downregulated genes considered for gene ontology analyses are in Table S1.

#### Error bars and associated p Values

All figures including error bars display mean with bars representing plus and minus the SEM(‘SEM’). p-values associated with error bars are determined using a two-sided Fisher exact test.

### Additional resources

#### Neural induction medium

• DMEM/F-12 (Caisson Labs, Cat No. DFL15)
• Knockout serum replacement (Thermo Fisher, Cat No. 10828-028) 15% (v/v)
• MEM-NEAA (Thermo Fisher, Cat No. 11140050) 1% (v/v)
• Glutamax supplement (Thermo Fisher, Cat No. 35050061) 1% (v/v)
• β-Mercaptoethanol (Sigma, Cat No. M7522) 100 μM
• LDN-193189 (Sigma, Cat No. SML0559) 100 nM
• SB431542 (Reprocell, Cat No. 04001005) 10 μM
• XAV939 (Stemgent, Cat No. 040046) 2 μM

#### Neural differentiation medium minus vitamin A

• DMEM/F-12 (Caisson Labs, Cat No. DFL15)
• Neurobasal medium (Thermo Fisher, Cat No. 12348017)
• Insulin (Santa Cruz, Cat No. sc-360248) 0.025% (v/v)
• MEM-NEAA (Thermo Fisher, Cat No. 11140050) 1% (v/v)
• Glutamax supplement (Thermo Fisher, Cat No. 35050061) 1% (v/v)
• Penicillin/Streptomycin (Caisson Labs, Cat No. PSL01) 1% (v/v)
• N2 supplement (Thermo Fisher, Cat No. 17502048) 0.5% (v/v)
• B27 supplement without vitamin A (Thermo Fisher, Cat No. 12587010) 1% (v/v)
• β-Mercaptoethanol (Sigma, Cat No. M7522) 50 μM

#### Neural differentiation medium

• DMEM/F-12 (Caisson Labs, Cat No. DFL15)
• Neurobasal medium (Thermo Fisher, Cat No. 12348017)
• Insulin (Santa Cruz, Cat No. sc-360248) 0.025% (v/v)
• MEM-NEAA (Thermo Fisher, Cat No. 11140050) 1% (v/v)
• Glutamax supplement (Thermo Fisher, Cat No. 35050061) 1% (v/v)
• Penicillin/Streptomycin (Caisson Labs, Cat No. PSL01) 1% (v/v)
• N2 supplement (Thermo Fisher, Cat No. 17502048) 0.5% (v/v)
• B27 supplement without vitamin A (Thermo Fisher, Cat No. 12587010) 1% (v/v)
• β-Mercaptoethanol (Sigma, Cat No. M7522) 50 μM
• BDNF (R&D Systems, Cat No. 248-BDB-050) 20 ng/ml
• Ascorbic acid (Tocris, Cat No. 4055) 200 μM

## Data Availability

• Single-cell RNA-seq data from hCOs have been deposited at GEO and are publicly available as of the date of publication (accession code GSE … …) • Bulk RNA-seq data from mouse cortices generated for this study have been similarly deposited or have previously been made available.• Newly reported sequencing data (P0/P1 for both mouse line and E13 for constitutive mouse model) can be accessed at accession code. • RNA-seq data for E13 for conditional mouse model initially generated for Sapir et al.^24^ was previously made available in the NCBI GEO database under accession code GSE181527. • No novel code is reported. All software required for the work within this manuscript (e.g. Seurat, Kallisto) is referenced in the key resources table and is publicly available.

## Supporting citations

The following references appear in the supplemental information: (^44^^,^^45^^,^^46^).

## Supplemental information

Document S1. Figures S1–S8 and Tables S2 and S3

Table S1. Upregulated and downregulated genes in *HNRNPU*^+/−^ hCOs, related to STAR Methods Pseudobulk analyses with MAST were used for both D11 and M20 and genes differentially expressed in same direction (FDR<.05, log_2_ fold change >0.25) were considered differentially expressed.
