# Supplementary material for: Evidence of shared transcriptomic dysregulation of HNRNPU-related disorder between human organoids and embryonic mice
Source: iScience. 2022 Dec 10;26(1):105797. doi: 10.1016/j.isci.2022.105797 (PMC9804147; doi:10.1016/j.isci.2022.105797)
Supplement: Document S1. Figures S1–S8 and Tables S2 and S3 [file mmc1.pdf]

iScience, Volume *26*

Supplemental information

Evidence of shared transcriptomic dysregulation of HNRNPU-related disorder between human organoids and embryonic mice

Andrew K. Ressler, Gabriela L.A. Sampaio, Sarah A. Dugger, Tamar Sapir, Daniel Krizay, Michael J. Boland, Orly Reiner, and David B. Goldstein

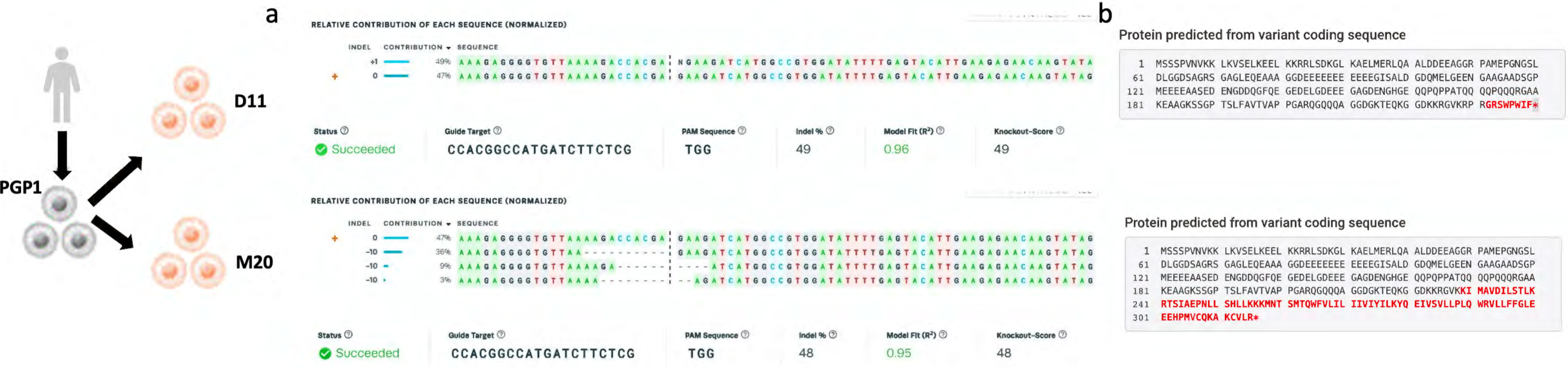

Supplementary Figure 1

a b

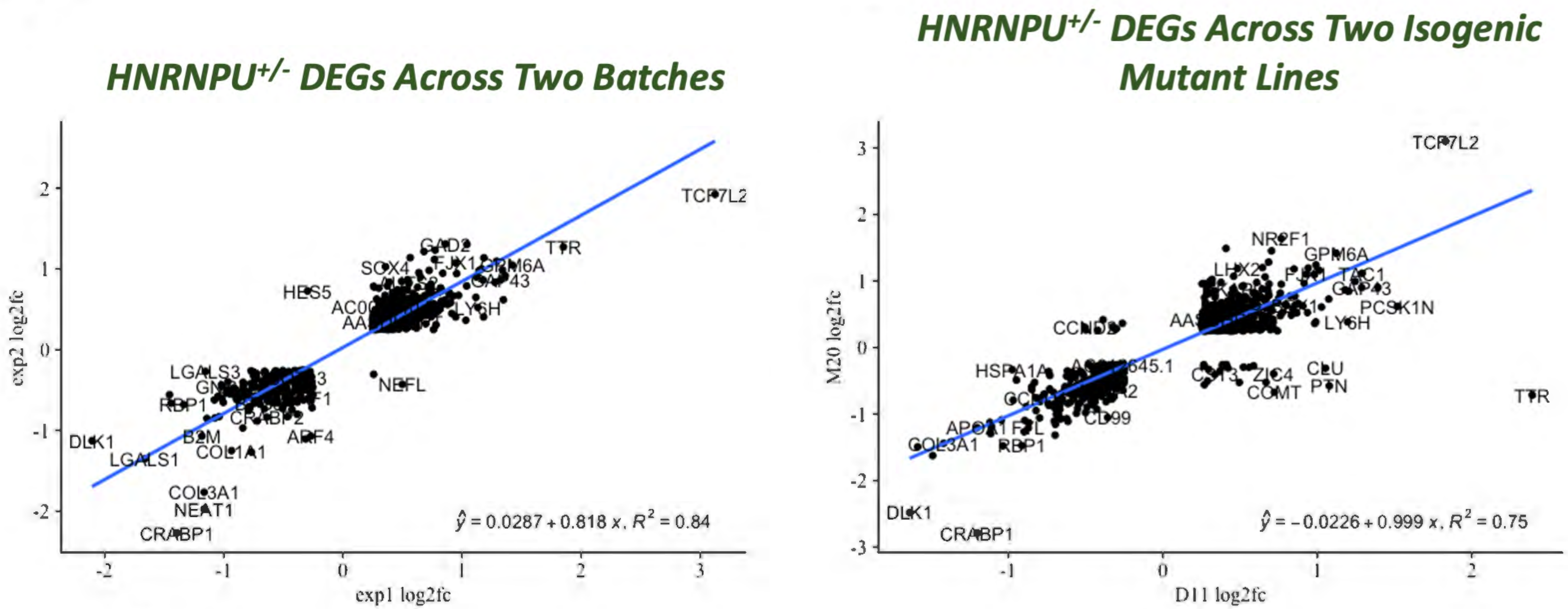

c

Enrichment DEGs	Expt1 vs Expt2		D11 vs M20	
	GME	***GME pval***	***GME***	***GME pval***
Up / Up	22.2	0	13.4	5.5e-237
Up / Down	.06	ns	.53	ns
Down / Up	.04	ns	.22	ns
Down / Down	42.3	1.5e-210	63.0	4.5e-250

Supplementary Figure 2

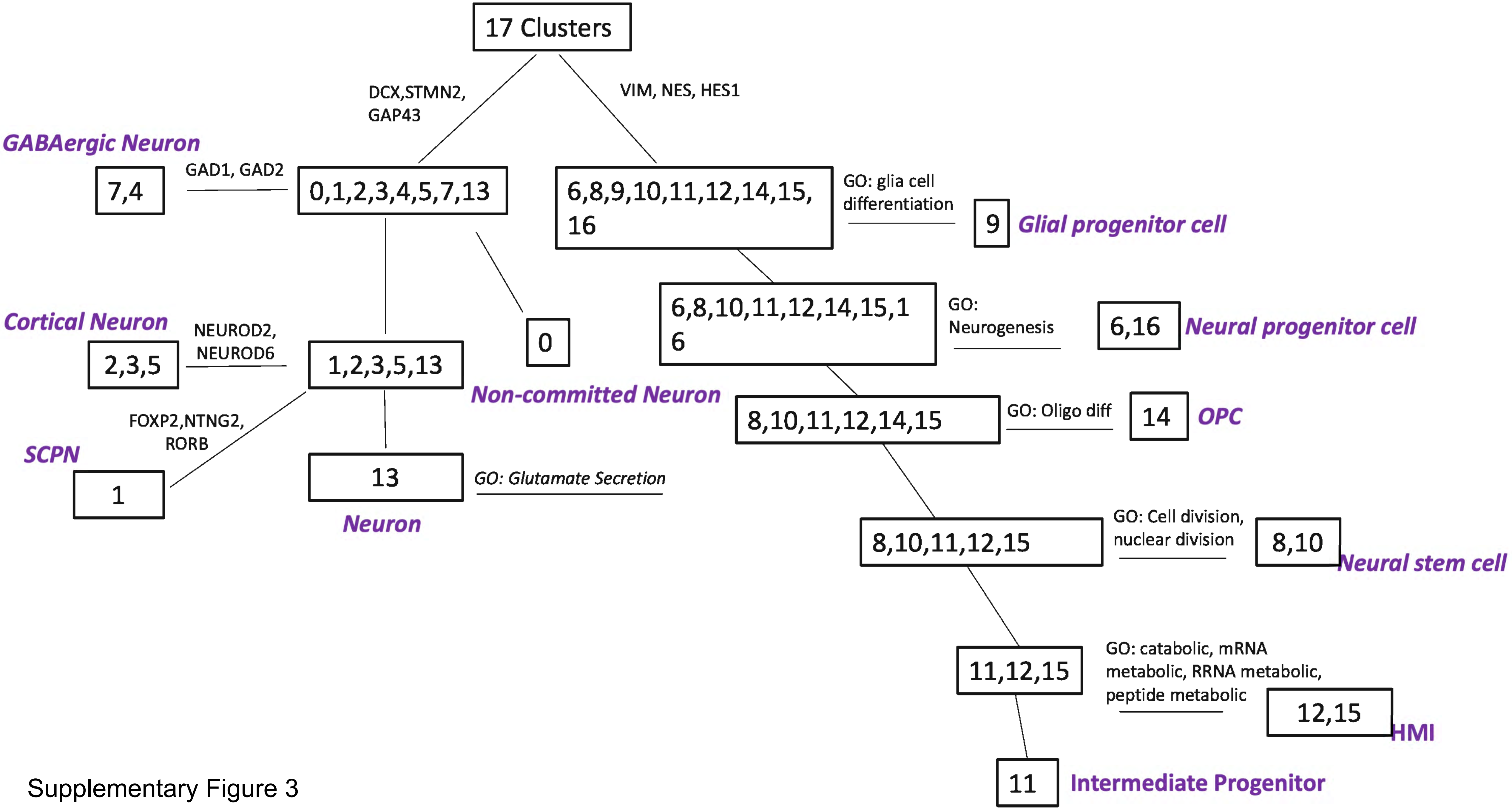

Supplementary Figure 3

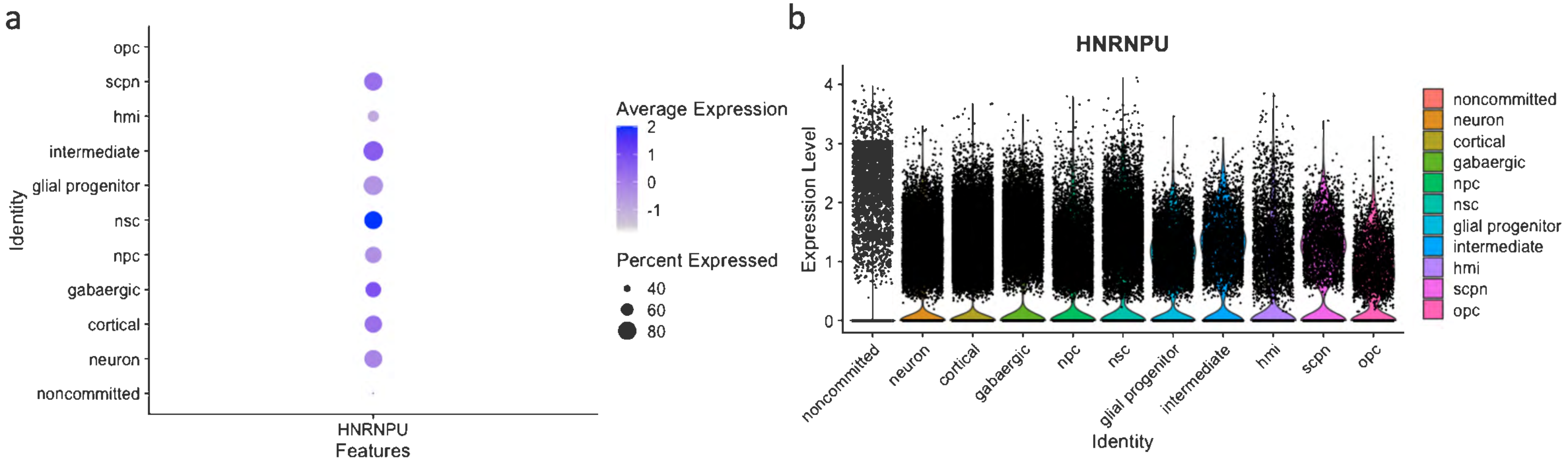

Supplementary Figure 4

Cluster	Condition 1	Condition 2	p	test use
noncommitted	D11	M20	0.150794	Wilcoxon
noncommitted	D11	PGP	0.222222	Wilcoxon
noncommitted	M20	PGP	0.309524	Wilcoxon
neuron	D11	M20	0.150794	Wilcoxon
neuron	D11	PGP	0.095238	Wilcoxon
neuron	M20	PGP	0.690476	Wilcoxon
cortical	D11	M20	0.150794	Wilcoxon
cortical	D11	PGP	0.055556	Wilcoxon
cortical	M20	PGP	0.690476	Wilcoxon
gabaergic	D11	M20	0.150794	Wilcoxon
gabaergic	D11	PGP	0.031746	Wilcoxon
gabaergic	M20	PGP	0.055556	Wilcoxon
npc	D11	M20	0.222222	Wilcoxon
npc	D11	PGP	0.007937	Wilcoxon
npc	M20	PGP	0.007937	Wilcoxon
nsc	D11	M20	0.309524	Wilcoxon
nsc	D11	PGP	0.222222	Wilcoxon
nsc	M20	PGP	0.095238	Wilcoxon
glial progenitor	D11	M20	0.150794	Wilcoxon
glial progenitor	D11	PGP	0.095238	Wilcoxon
glial progenitor	M20	PGP	0.690476	Wilcoxon
intermediate	D11	M20	0.690476	Wilcoxon
intermediate	D11	PGP	1	Wilcoxon
intermediate	M20	PGP	0.150794	Wilcoxon
hmi	D11	M20	0.007937	Wilcoxon
hmi	D11	PGP	0.007937	Wilcoxon
hmi	M20	PGP	0.007937	Wilcoxon
scpn	D11	M20	0.420635	Wilcoxon
scpn	D11	PGP	0.055556	Wilcoxon
scpn	M20	PGP	0.007937	Wilcoxon
opc	D11	M20	0.007937	Wilcoxon
opc	D11	PGP	0.222222	Wilcoxon
opc	M20	PGP	0.007937	Wilcoxon

Supplementary Table 2

a

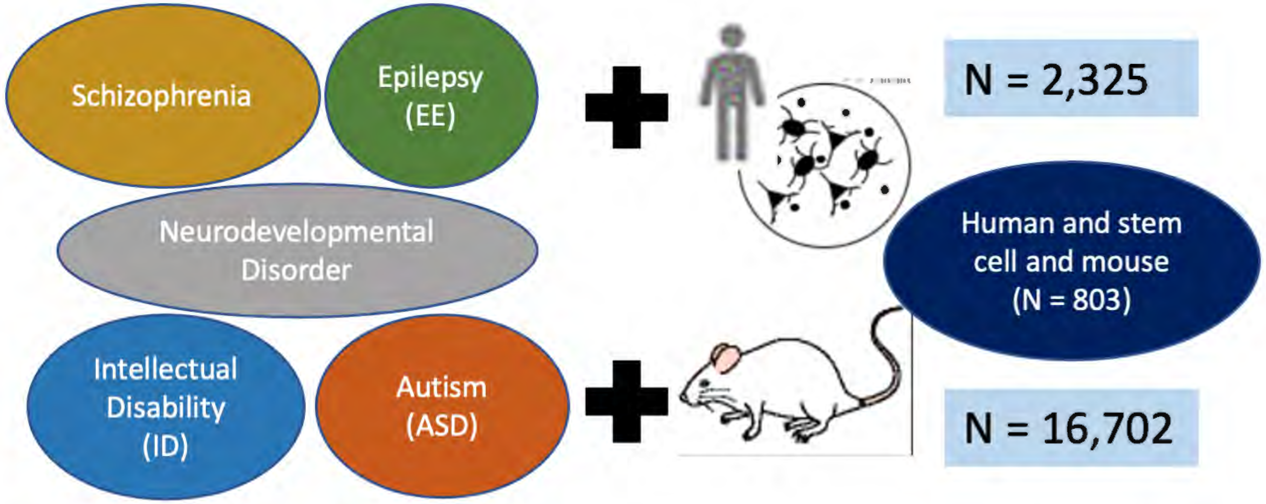

b

Paper Title	Mouse Ages	Human Neurons	Mouse vs Human Results
Bioinformatics Analyses of the Transcriptome Reveal Ube3a-Dependent Effects on Mitochondrial-Related Pathways (Panov 2020)	3-4 Months	Neurons 10 weeks – EBs/Monolayer	'Mouse and Human Recapitulate Disruption of Mitochondrial Gene Dysregulation'
Temporal Control of Mammalian Cortical Neurogenesis by m(6)A Methylation (Yoon 2017)	E13.5	Organoids d47	'Conserved and Unique Features of Human m6A Landscape during Cortical Neurogenesis'
Transcriptional consequences of MBD5 disruption in mouse brain and CRISPR-derived neurons (Seabra 2020)	8 weeks	NPC and Neurons (10 passages)	'lack of significant overlap of the latter (neuron) DEGs with the mouse brain results'

Supplementary Figure 5

Enrichment DEGs	*HNRNPU*$^{+/-}$ hCOs vs Hnrnpu$^{fl/-}$ E13 Cortices	
	GME	***GME pval***
Up *HNRNPU*$^{+/-}$ hCOs / Up *Hnrnpu*$^{fl/-}$ E13	2.9	1.1e-27
Up *HNRNPU*$^{+/-}$ hCOs / Down *Hnrnpu*$^{fl/-}$ E13	.77	ns
Down *HNRNPU*$^{+/-}$ hCOs / Up *Hnrnpu*$^{fl/-}$ E13	.35	ns
Down *HNRNPU*$^{+/-}$ hCOs / Down *Hnrnpu*$^{fl/-}$ E13	3.4	4.1e-19

Supplementary Table 3

Hnrnpu+/- E13 (172 DEGs)	*Hnrnpu*fl/- E13 (2,802 DEGs) and *Hnrnpu*fl/fl E13

a

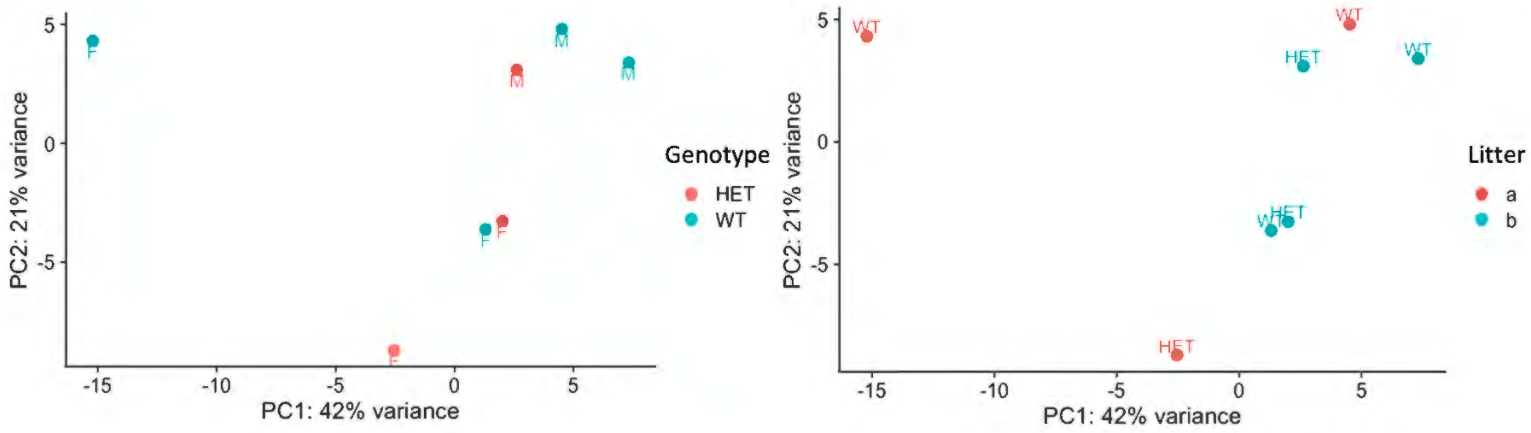

b

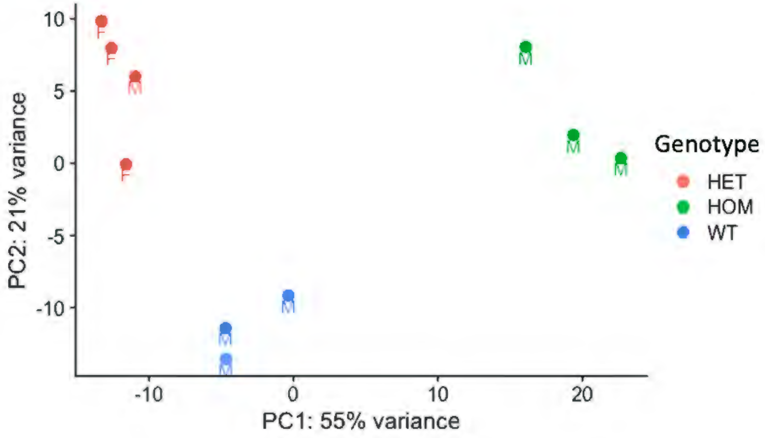

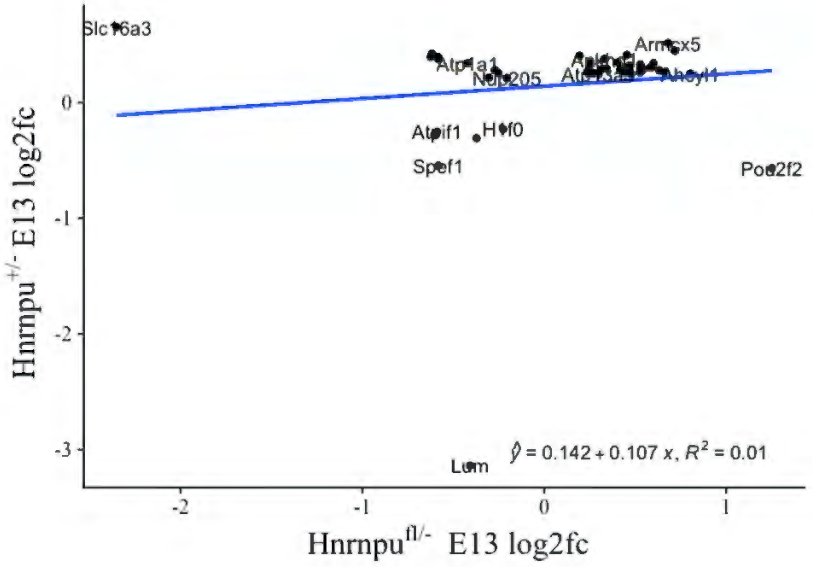

	*Hnrnpu*fl/- / *Hnrnpu*+/-	P-value	Odds
1	Up *Hnrnpu*fl/- / up *Hnrnpu*+/-	1.5e-6	3.1
2	Up *Hnrnpu*fl/- / down Hnrnpu+/-	ns	.23
3	Down *Hnrnpu*fl/- / up *Hnrnpu*+/-	ns	1.2
4	Down *Hnrnpu*fl/- / down *Hnrnpu*+/-	ns	1.5

Supplementary Figure 6

a

HNRNPU+/- hCOs vs P1 Heterozygotes — *HNRNPU+/- hCOs vs E13 Homozygotes* — *HNRNPU+/- hCOs vs P1 Homozygotes*

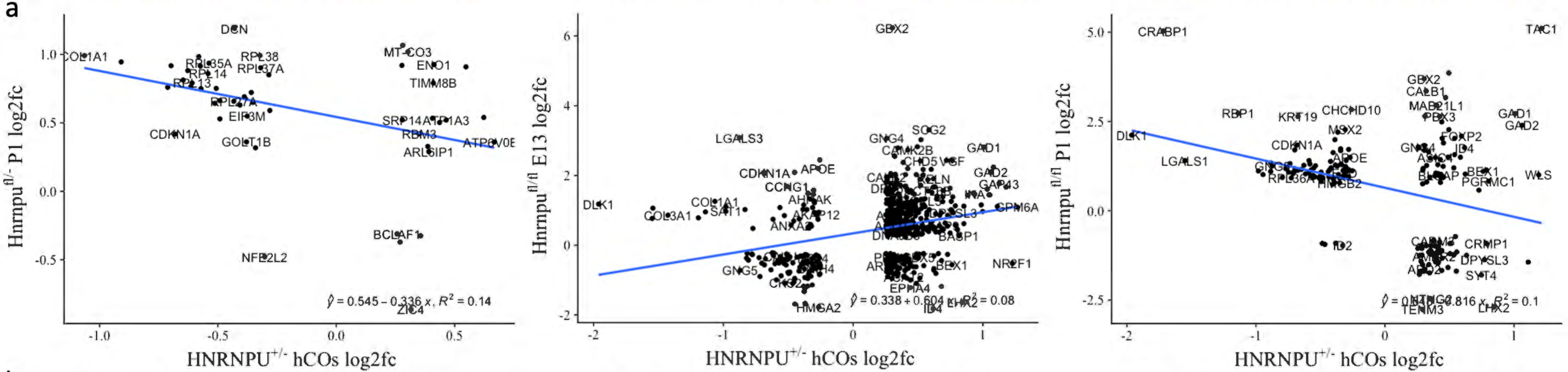

b

hCOs / P1 Hnrnpu$^{fl/-}$	p-value	GME
Up *HNRNPU+/-* hCOs / up *Hnrnpu$^{fl/-}$*	ns	1.5
Up *HNRNPU+/-* hCOs / down *Hnrnpu$^{fl/-}$*	ns	.50
Down *HNRNPU+/-* hCOs / up *Hnrnpu$^{fl/-}$*	6.2e-16	7.7
Down *HNRNPU+/-* hCOs / down *Hnrnpu$^{fl/-}$*	ns	.28

hCOs / E13 *Hnrnpu$^{fl/fl}$*	p-value	GME
Up *HNRNPU+/-* hCOs / up *Hnrnpu$^{fl/fl}$*	8.7e-60	3.9
Up *HNRNPU+/-* hCOs / down *Hnrnpu$^{fl/fl}$*	ns	.61
Down *HNRNPU+/-* hCOs / up *Hnrnpu$^{fl/fl}$*	ns	.76
Down *HNRNPU+/-* hCOs / down *Hnrnpu$^{fl/fl}$*	2.6e-2	1.3

hCOs / P1 Hnrnpu$^{fl/fl}$	p-value	GME
Up *HNRNPU+/-* hCOs / up *Hnrnpu$^{fl/fl}$*	1.8e-2	1.4
Up HNRNPU+/- hCOs / down *Hnrnpu$^{fl/fl}$*	5.7e-4	1.7
Down *HNRNPU+/-* hCOs / up *Hnrnpu$^{fl/fl}$*	1.9e-28	5.8
Down *HNRNPU+/-* hCOs / down *Hnrnpu$^{fl/fl}$*	ns	.28

Supplementary Figure 7

a

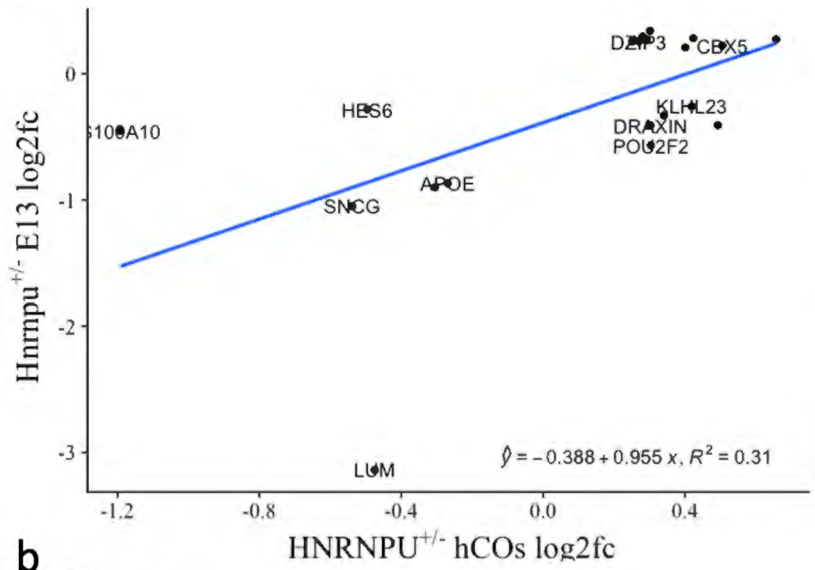

***HNRNPU*$^{+/-}$ hCOs / *Hnrnpu*$^{+/-}$ E13**	**P-value**	**Odds**
Up *HNRNPU*$^{+/-}$ hCOs / up *Hnrnpu*$^{+/-}$ E13	ns	1.4
Up *HNRNPU*$^{+/-}$ hCOs / down *Hnrnpu*$^{+/-}$ E13	ns	2.3
Down *HNRNPU*$^{+/-}$ hCOs / up *Hnrnpu*$^{+/-}$ E13	ns	0
Down *HNRNPU*$^{+/-}$ hCOs / down *Hnrnpu*$^{+/-}$ E13	0.00082	6.2

b

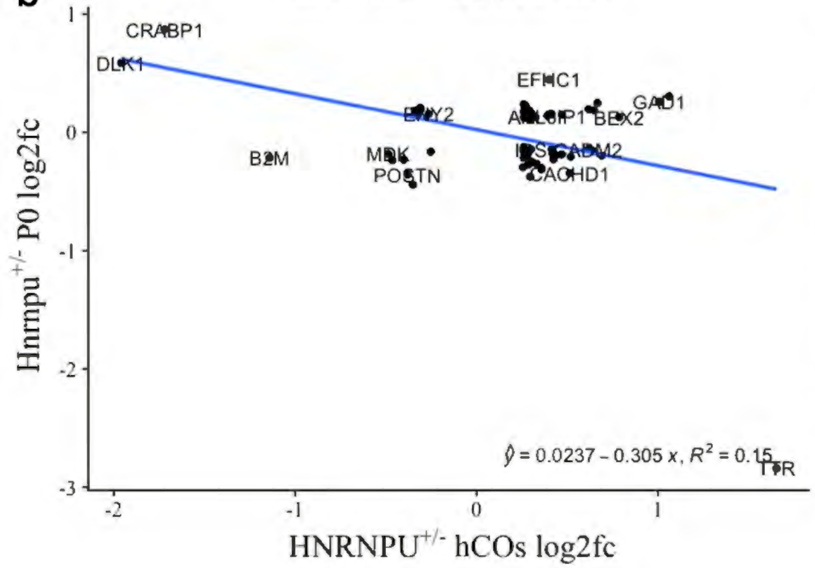

***HNRNPU*$^{+/-}$ hCOs / *Hnrnpu*$^{+/-}$ E13**	**P-value**	**Odds**
Up *HNRNPU*$^{+/-}$ hCOs / up *Hnrnpu*$^{+/-}$ P0	ns	0.90
Up *HNRNPU*$^{+/-}$ hCOs / down *Hnrnpu*$^{+/-}$ P0	ns	0.85
Down *HNRNPU*$^{+/-}$ hCOs / up *Hnrnpu*$^{+/-}$ P0	ns	0.63
Down *HNRNPU*$^{+/-}$ hCOs / down *Hnrnpu*$^{+/-}$ P0	ns	0.56

c

Assessment of 31 genes downregulated in organoids and upregulated in *Hnrnpu*$^{fl/-}$; *Emx1*-Cre P1 cortices

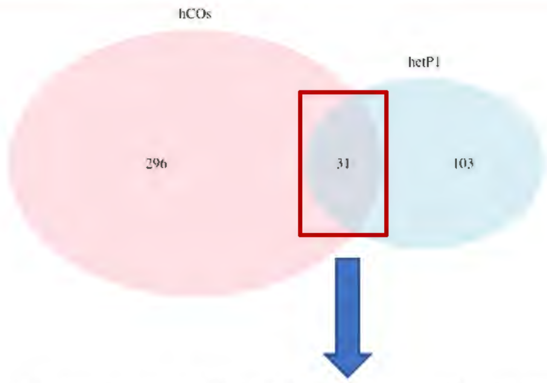

Histogram of log2fc of discordant genes in *Hnrnpu*$^{+/-}$ P0 cortices

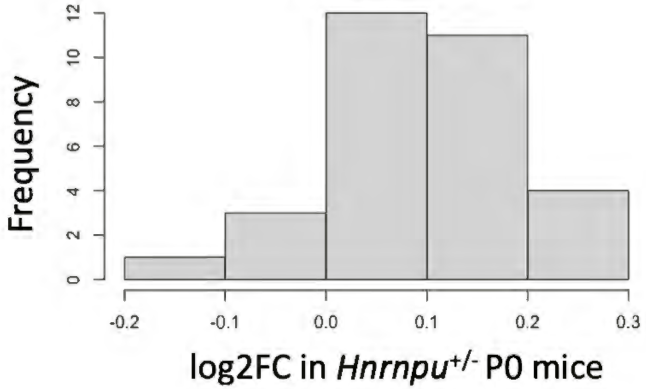

Direction of log2fc in *Hnrnpu*$^{+/-}$ P0 mice

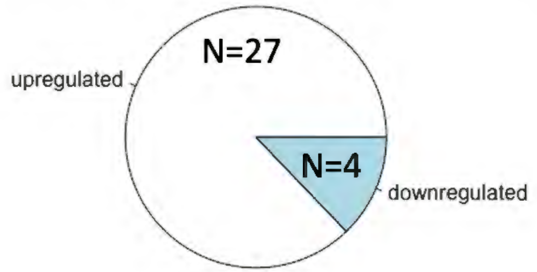

Supplementary Figure 8

Supplementary information legends

Supplementary figure 1: *Generation of HNRNPU$^{+/-}$ knockout stem cell lines*. Both D11 and M20 cell lines were generated using CRISPR-Cas9 and cut location Chr: 1; 244,862,672, which is within Exon 2 of *HNRNPU*. a) D11 has a heterozygous 1 bp duplication and M20 has a heterozygous 10 bp deletion confirmed by Sanger sequencing by Synthego^{TM}. b) Predicted variant coding sequence using Mutalyzer (https://mutalyzer.nl/). Both variants lead to premature stop-codons in exons 2 (D11) and 4 (M20) respectively. Related to STAR Methods.

Supplementary figure 2: *HNRNPU$^{+/-}$ hCOs transcriptomic signature reproduced across experiments and isogenic mutant lines.* a) and b) Scatter plot of log2fc of significant *HNRNPU*$^{+/-}$ DEGs (FDR<.05) between two experiments (n=18 organoids (expt1) and 24 organoids (expt2)) and two isogenic mutant lines, D11 and M20 versus PGP1 controls (n=42 organoids each). c) Geometric mean enrichment of gene sets between experiments and isogenic mutant lines shows highly significant enrichment of co-upregulated and co-downregulated genes. Related to STAR Methods.

Supplementary figure 3: *Annotation of clusters in DIV45 hCOs*. Clusters first separated into neuronal clusters by DCX, STMN2, GAP43 and proliferative and cortical precursor cells by VIM, NES and HES1. Cluster 0 was predominantly positive for neuronal markers but showed no specific enrichment of neuronal subtype markers or subtype specific neurotransmitters and were thus labeled as "noncommitted." All other clusters were classified using enrichment of canonical markers and gene ontologies. Enriched genes generated using MAST conserved markers across D11, M20 and PGP1. 'SCPN '= sub-cortical projection neuron. 'OPC '= Oligodendrocyte progenitor cell. 'HMI '= High metabolic intermediate progenitor cell. Related to STAR Methods.

Supplementary figure 4: *hnRNPU expression in DIV45 hCOs.* Average Expression of hnRNPU in annotated cell clusters shown using Seurat's a) DotPlot and b) Violin Plot functions. For dot plot, size of circle shows percent of cells expressing hnRNPU mRNA transcripts, while the coloring shows average expressing. Average expression highest in NSCs, but no clear patterns of expression across cell types and hnRNPU is expressed in all cell types. Related to STAR Methods and figure 2.

Supplementary table 2: *Significance testing of compositional differences in HNRNPU$^{+/-}$ hCOs.* For each line, all five samples treated as independent. Directionally consistent significant or trending significant (<.05 or <.06) differences between *HNRNPU*$^{+/-}$ and PGP1 for GABAergic, NPC, HMI and SCPN populations. Related to figure 2.

Supplementary figure 5: *Cross-species trancriptomic and epitranscriptomic comparisons.* Literature search shows few attempts to compare transcriptomic or epitranscriptomic signature across human stem cell and mouse models of neurodevelopmental disorders. a) Searched for all papers that mentioned one of several descriptors of neurodevelopmental disorders and found 803 papers that include all three terms human, stem cell and mouse. b) Manual examination of 803 papers identified three papers[44,45,46] with modestly robust comparison of dysregulated genes across species. Importantly, only two considered transcriptomic or epitranscriptomic patterns across all gene sets. Yoon et al. found consistent methylation signatures when looking at

embryonic mice and 47-day-old organoids, while Seabra et al. did not find significant overlap looking at 8-week-old mice compared to a human *in vitro* system, providing limited evidence that consideration of developmentally matched time points may be informative. Related to STAR Methods.

Supplementary table 3: *HNRNPU$^{+/-}$ hCOs and Hnrnpu$^{fl/-}$ E13 cortices show significant enrichment of co-dysregulated genes*. Geometric mean enrichment ('GME') and corresponding p-values ('pval') are shown for all combinations of up and downregulated genes. Related to figure 3.

Supplementary figure 6: *Enrichment of co-upregulated genes in two embryonic mouse models of HNRNPU-related disorder.* a) Cortical bulk RNA-seq of Constitutive knockout of *Hnrnpu*$^{+/-}$ results in 172 DEGs (FDR <.05, DESeq2), but no evidence of clustering in PC space by genotype or litter, while samples do cluster by gender. b) In heterozygous mice with a conditional truncating mutation in *Hnrnpu* (*Hnrnpu*$^{fl/-}$ or *Hnrnpu*$^{fl/fl}$), cortical bulk RNA-seq shows clear stratification by genotype. c) No enrichment of sign mismatched gene sets between heterozygous mice from the two distinct mouse models, while there is ~3-fold enrichment of co-upregulated genes, elucidating some overlap of transcriptomic dysregulation across mouse models. "F" = female, "M" = male. Related to STAR Methods and figure 3.

Supplementary figure 7: *Downregulated genes in HNRNPU$^{+/-}$ hCOs upregulated in perinatal Hnrnpu$^{fl/-}$ and Hnrnpu$^{fl/fl}$ cortices.* a) Best fit linear regression line shows evidence of negative correlation of log2 fold change of gene correlates in *HNRNPU*$^{+/-}$ hCOs and perinatal heterozygous (left) and homozygous (right) conditional knockouts, while *HNRNPU*$^{+/-}$ dysregulation is positively correlated with embryonic homozyogotes (middle). b) Both heterozygous (left) and homozygous (right) perinatal cortices show significant upregulation of genes downregulated in in *HNRNPU*$^{+/-}$ hCOs. Alternatively, embryonic homozyogtes (middle) show enrichment in both co-upregulated and co-downregulated genes, without any evidence of enrichment in sign reversed gene sets, suggesting partial reproduction of transcriptomic dysregulation across models. For analyses of perinatal heterozygotes, unadjusted p-values <.05 used for differential expression due to limited number of FDR-adjusted DEGs. Related to figure 4.

Supplementary figure 8: *Developmental divergence of transcriptomic signature evident in Hnrnpu$^{+/-}$ mice.* a) Positive correlation (R2 = .31) and significant ~6-fold enrichment in co-downregulated genes between *Hnrnpu*$^{+/-}$ E13 and DIV45 *HNRNPU*$^{+/-}$ hCO DEGs. b) Negative slope (R2 = .15) and no evidence of significantly enriched discordant or concordant gene sets between DIV45 *HNRNPU*$^{+/-}$ DEGs and *Hnrnpu*$^{+/-}$ P0 DEGs (unadjusted pval<.05 use for P0 due to absence of genes with FDR-adjusted significance). c) Genes downregulated in hCOs and upregulated in perinatal *Hnrnpu*$^{fl/-}$ cortices predominantly upregulated in perinatal *Hnrnpu*$^{+/-}$ cortices (binomial distribution, p<.00001). Related to figure 4.
